# Macrophage depletion protects against cisplatin-induced ototoxicity and nephrotoxicity

**DOI:** 10.1126/sciadv.adk9878

**Published:** 2024-07-24

**Authors:** Cathy Yea Won Sung, Naoki Hayase, Peter S. T. Yuen, John Lee, Katharine Fernandez, Xuzhen Hu, Hui Cheng, Robert A. Star, Mark E. Warchol, Lisa L. Cunningham

**Affiliations:** ^1^ Laboratory of Hearing Biology and Therapeutics, National Institute on Deafness and Other Communication Disorders (NIDCD), NIH, Bethesda, MD, USA.; ^2^ Renal Diagnostics and Therapeutics Unit, National Institute of Diabetes and Digestive and Kidney Diseases (NIDDK), NIH, Bethesda, MD, USA.; ^3^ Bioinformatics and Biostatistics Collaboration Core, National Institute on Deafness and Other Communication Disorders (NIDCD), NIH, Bethesda, MD, USA.; ^4^ Department of Otolaryngology, School of Medicine, Washington University, Saint Louis, MO, USA.

## Abstract

Cisplatin is a widely used anticancer drug with notable side effects including ototoxicity and nephrotoxicity. Macrophages, the major resident immune cells in the cochlea and kidney, are important drivers of both inflammatory and tissue repair responses. To investigate the roles of macrophages in cisplatin-induced toxicities, we used PLX3397, a U.S. Food and Drug Administration–approved inhibitor of the colony-stimulating factor 1 receptor, to eliminate tissue-resident macrophages. Mice treated with cisplatin alone had considerable hearing loss (ototoxicity) and kidney injury (nephrotoxicity). Macrophage ablation resulted in significantly reduced hearing loss and had greater outer hair cell survival. Macrophage ablation also protected against cisplatin-induced nephrotoxicity, as evidenced by markedly reduced tubular injury and fibrosis. Mechanistically, our data suggest that the protective effect of macrophage ablation against cisplatin-induced ototoxicity and nephrotoxicity is mediated by reduced platinum accumulation in both the inner ear and the kidney. Together, our data indicate that ablation of tissue-resident macrophages represents an important strategy for mitigating cisplatin-induced ototoxicity and nephrotoxicity.

## Teaser

Macrophage ablation protects against ototoxicity and nephrotoxicity by limiting cisplatin accumulation in the cochlea and kidney.

## INTRODUCTION

Cisplatin is a platinum-based chemotherapeutic drug that is widely used and highly effective in treating a variety of solid tumors, including testicular, bladder, lung, stomach, head and neck, and ovarian cancers, in both pediatric and adult patients (*1*). It exerts its antitumor activity by forming DNA cross-links that interfere with replication, transcription, and DNA repair mechanisms, causing DNA damage and subsequent apoptosis in cancer cells (*1*). Cisplatin therapy is associated with notable side effects, including ototoxicity, nephrotoxicity, and myelosuppression, which can be dose-limiting and can compromise therapeutic outcomes as well as quality of life for cancer survivors (*2*, *3*).

Cisplatin is the most ototoxic drug in clinical use, resulting in bilateral, progressive, and permanent sensorineural hearing loss in up to 60% of treated adults (*4*, *5*) and 70% of treated children (*6*). Cisplatin results in the death of mechanosensory hair cells in the inner ear that are required for hearing function (*7*). Recently, the U.S. Food and Drug Administration (FDA) approved the use of sodium thiosulfate as an otoprotective agent for mitigating cisplatin-induced ototoxicity in pediatric patients with localized, nonmetastatic solid tumors (*8*). Despite the considerable progress in ototoxicity research, there are currently no effective treatment strategies for cisplatin-induced ototoxicity in adults or in children with metastatic cancers. In addition to ototoxicity, cisplatin also results in kidney damage and renal dysfunction. Approximately 30 to 40% of patients with cancer treated with cisplatin develop acute kidney injury (*9*). In addition, patients without acute kidney injury remain susceptible to the development of chronic kidney disease both during and after the discontinuation of cisplatin treatment. Currently, there are no effective pharmacological treatments available for patients with cancer receiving cisplatin-based therapies who develop chronic kidney disease without acute kidney injury (*10*). Thus, there is an unmet clinical need for innovative treatments that limit or prevent cisplatin-induced ototoxicity and nephrotoxicity.

**Fig. 1.**
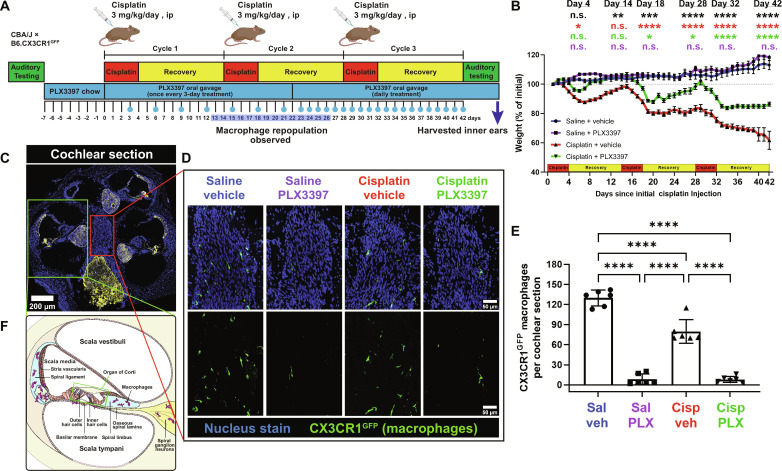
Initial protocol in experiment 1 resulted in macrophage ablation via PLX3397, followed by partial macrophage repopulation. (**A**) Experimental design showing auditory tests, PLX3397 treatment, and 3 cycles of cisplatin administration. Mice received PLX3397-formulated chow for 7 days to facilitate macrophage depletion. During cisplatin administration, mice received PLX3397 via oral gavage once every 3 days. Upon observation of macrophage repopulation on day 14 (see fig. S1), daily treatment of PLX3397 via oral gavage was initiated. The days on which mice received PLX3397 via oral gavage are denoted with blue circles. ip, intraperitonally. (**B**) Changes in mouse weight are shown throughout the 3 cycles of cisplatin administration protocol. Means ± SEM, *n* = 10 to 16 mice per experimental group (total 49 mice; 24 females and 25 males). Statistical comparisons (asterisks or n.s.) are color-coded as described in Methods. (**C**) Cochleae harvested after end-point auditory tests were immunolabeled for Kir4.1 (yellow) to visualize cochlear structures and green fluorescent protein (GFP) to visualize CX3CR1^GFP^-positive macrophages (green). Statistical significance is indicated as the following: *P < 0.05, **P < 0.01, ***P < 0.001, and ****P < 0.0001. *P* value above 0.05 (*P* > 0.05) was considered nonsignificant (n.s.). (**D**) Representative confocal images [cochlear modiolus; from red box in (C)] and (**E**) quantification of macrophages per cochlear section. PLX3397 resulted in >93% depletion of all macrophages. (**F**) Schematic diagram of macrophage distribution in the middle cochlear turn [from green box in (C)]. Scale bars, 200 μm (cochlear section) and 50 μm (mid-modiolar section). Means ± SD, *n* = 6 cochleae (3 females and 3 males) per experimental group (total 24 cochleae; 12 females and 12 males). One-way analysis of variance (ANOVA) with Tukey’s multiple comparisons test.

Cochlear macrophages are the major innate immune cells in the cochlea and act as important drivers of both inflammatory (*11*) and tissue repair responses (*12*) after cochlear injury. Macrophages account for >95% of CD45^+^ leukocytes in the adult mouse cochlea (*13*) and are also found in large numbers in the human cochlea (*14*). Under steady-state conditions, macrophages are present in the osseous spiral lamina, basilar membrane, spiral ganglion neurons (SGNs), spiral ligament, and stria vascularis (*13*), while the organ of Corti is mostly devoid of macrophages (Fig. 1F and figs. S2C and S6C) (*13*). The stria vascularis contains the blood supply of the cochlea and a unique subset of tissue-resident macrophages, the perivascular macrophages (PVMs), that are closely associated with the blood vessels and function as part of the blood-labyrinth barrier (BLB) (*15*, *16*). The BLB between the lumen of the vasculature and the inner ear fluid spaces tightly controls the exchange of substances circulating in the bloodstream and those in the inner ear (*17*). PVMs are important for BLB function and regulate the BLB permeability through either direct physical interaction with the capillaries or by secreting soluble factors that act on vascular endothelial cells that line the capillary lumen (*15*, *18*). Insults to the cochlea, such as exposure to loud noise (*19*, *20*), bacterial or viral infections (*21*, *22*), or insertion of a cochlear implant (*23*), can activate tissue-resident macrophages, which can up-regulate inflammatory cytokines, phagocytose tissue debris, or induce infiltration of peripheral immune cells to the site of injury (*24*). However, the roles of cochlear macrophages in cisplatin-induced ototoxicity are unknown.

Kidney-resident macrophages are critical components of the innate immune system in the kidney, and they are involved in maintaining tissue homeostasis, immune surveillance, response to tissue injury, and wound healing (*25*). Kidney-resident macrophages account for ~50 to 60% of CD45^+^ leukocytes in the mouse kidney (*26*) and include heterogeneous populations with varying phenotypes and functions depending on their location within the kidney (*27*). Kidney-resident macrophages are found closely associated with capillaries that surround the tubules and glomeruli (*26*) and are involved in the regulation of tubular epithelial cell function or induction of apoptosis during tubulointerstitial inflammation (*28*). Moreover, emerging evidence suggests that kidney-resident macrophages are involved in mediating cisplatin-induced renal injury (*29*). However, the precise mechanisms by which renal macrophages contribute to cisplatin-induced nephrotoxicity are not fully understood.

The goals of the present study were to examine the roles macrophages play in cisplatin-induced ototoxicity and nephrotoxicity and to identify the mechanism(s) underlying their activity in response to cisplatin using a mouse model of cisplatin ototoxicity that results in hearing loss that is similar to that observed clinically (*30*). We used a pharmacological inhibitor of colony-stimulating factor 1 receptor (CSF1R) to achieve prolonged depletion of tissue-resident macrophages in vivo. CSF1 signaling through its receptor CSF1R promotes proliferation, differentiation, and survival of tissue-resident macrophages (*31*). PLX3397 is a small-molecule inhibitor of CSF1R that penetrates the blood-brain barrier and results in the elimination of 95 to 99% of all microglia in the central nervous system (CNS) (*31*, *32*). PLX3397-induced macrophage/microglia ablation in the CNS is achieved while minimally affecting circulating monocytes or other immune cells (*33*). We administered PLX3397 7 days before and throughout the cisplatin administration period to examine the roles of macrophages during cisplatin treatment. Our data indicated that >88% of tissue-resident macrophages were eliminated in both the cochlea and the kidney after PLX3397 treatment. Macrophage ablation resulted in significant protection against cisplatin-induced hearing loss and kidney injury, in part by limiting cisplatin accumulation in the cochlea and the kidney. Our findings strongly suggest that tissue-resident macrophages contribute to cisplatin entry and accumulation into the cochlea and the kidney. We propose macrophage-based immunotherapy as an approach to preventing cisplatin ototoxicity and nephrotoxicity in patients with cancer undergoing cisplatin therapy.

## RESULTS

### Incomplete macrophage ablation reduces cisplatin-induced ototoxicity (development of the cochlear macrophage ablation protocol)

Macrophages are the primary resident immune cells in the cochlea and play important roles in the response to cochlear injury (*12*, *15*). To investigate the roles of macrophages in cisplatin-induced ototoxicity, we ablated macrophages by pharmacologically inhibiting CSF1R signaling. Tissue-resident macrophages and microglia are highly dependent on CSF1R signaling for their survival (*31*). PLX3397 is a CSF1R antagonist and an inhibitor for c-Kit and FLT3 that penetrates the blood-brain barrier and results in elimination of 95 to 99% of all microglia in the CNS (*31*, *32*). The discontinuation of PLX3397 leads to robust repopulation of brain microglia within a 7-day period (*32*). While PLX3397 administration via chow feeding effectively ablates cochlear macrophages, mice receiving cisplatin demonstrate decreased appetite, potentially resulting in insufficient drug administration. To account for this, we fed mice with control or PLX3397-formulated chow for 7 days before cisplatin treatment, followed by PLX3397 (50 mg/kg) administration via oral gavage during the cisplatin administration protocol, and until mice were euthanized. Initially, PLX3397 was administered by oral gavage once every 3 days (Fig. 1A). This experimental design will be referred to as “experiment 1.” The inner ears were examined at 9, 14, and 28 days after the first cisplatin injection. At day 9, macrophages were depleted; however, by day 14, some macrophage repopulation was observed (fig. S1). Therefore, we adjusted the PLX3397 administration frequency, starting on day 22 of the 42-day protocol, from once every 3 days to daily treatment throughout the remainder of the cisplatin administration protocol. At day 28, following five consecutive days of daily PLX3397 administration, a significant and sustained reduction in cochlear macrophage population was observed (fig. S1). At the end of the experiment, mice that received PLX3397 exhibited depletion of ~93% of all CX3CR1^GFP^-labeled resident macrophages compared to saline/vehicle-treated mice (Fig. 1, D and E, and fig. S1). The depletion of macrophages was consistent across different compartments of the cochlea, including Rosenthal’s canal and modiolus (fig. S2, A, B, and D). Consistent with the literature, macrophages were not observed in the organ of Corti (fig. S2C) (*13*). Note that administration of cisplatin alone (cisplatin/vehicle) resulted in a 42.15% reduction in the number of cochlear macrophages compared to saline/vehicle-treated mice (Fig. 1, D and E). Saline-treated mice continued to gain weight throughout the experimental protocol, while all cisplatin-treated mice lost weight. Notably, mice treated with cisplatin/PLX3397 showed significantly reduced weight loss when compared to those treated with cisplatin/vehicle (Fig. 1B).

**Fig. 2.**
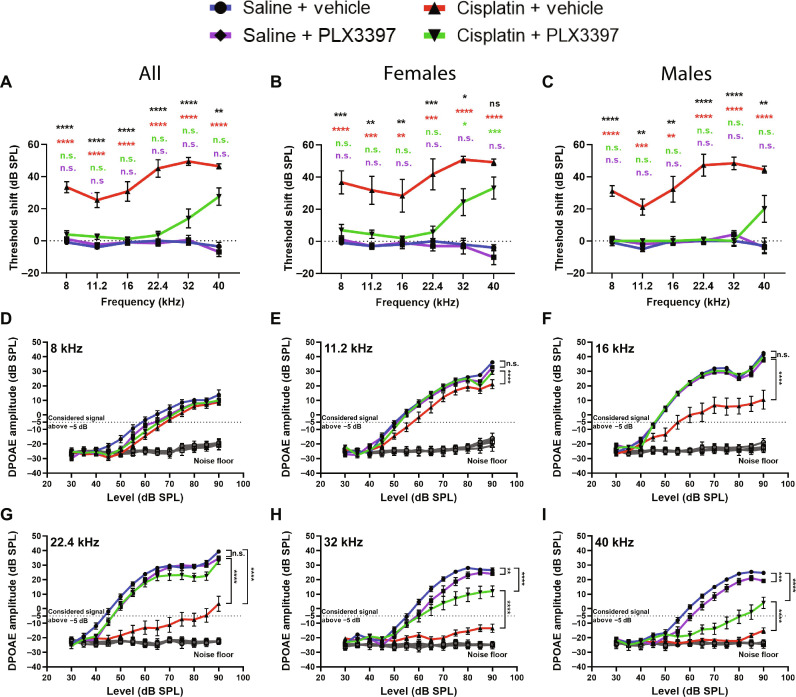
Macrophage ablation followed by partial repopulation protected against cisplatin-induced hearing loss and OHC dysfunction (experiment 1). (**A** to **C**) Auditory sensitivity was measured via ABRs at baseline and end point. Threshold shifts are reported as the difference between baseline and end point ABR thresholds [saline/vehicle (blue line), saline/PLX3397 (purple line), cisplatin/vehicle (red line), and cisplatin/PLX3397 (green line)]. (B and C) PLX3397 protected against cisplatin-induced hearing loss in both (B) female and (C) male mice, with better protection in males than in female (*P = 0.0277). *P* values were calculated using one-way ANOVA with Tukey’s multiple comparisons test. Means ± SEM, *n* = 10 to 16 mice per experimental group (5 to 7 females and 5 to 9 males). Statistical comparisons (asterisks or n.s.) are color-coded as described in Methods. (**D** to **I**) OHC function was measured via DPOAEs. An emission at 2*f*_1_ − *f*_2_ was considered present when its amplitude was greater than −5 dB (dotted lines). Statistical analyses were performed using two-way ANOVA with Tukey’s multiple comparisons test (main column effect). Means ± SEM, *n* = 10 to 16 mice (5 to 7 females and 5 to 9 males) per experimental group (total 49 mice; 24 females and 25 males).

To examine whether macrophages contributed to cisplatin-induced hearing loss, we measured auditory brainstem responses (ABRs) in control mice and in mice subjected to macrophage ablation via PLX3397. The auditory tests were performed before PLX3397 pretreatment (baseline) and at the end of the cisplatin administration protocol (end point). Differences in ABR thresholds between the end point and baseline measurements were calculated and reported as ABR threshold shifts. In the absence of cisplatin, PLX3397 treatment resulted in no significant changes in hearing sensitivity (Fig. 2, A to C). Consistent with our previous results (*7*, *30*), all cisplatin-treated mice exhibited significant increases in ABR thresholds compared to baseline. Notably, mice treated with both cisplatin and PLX3397 exhibited significantly reduced ABR threshold shifts compared to mice treated with cisplatin alone (cisplatin/vehicle) (Fig. 2A). The protective effect of PLX3397 was observed in both cisplatin-treated female and male mice, with greater protection observed in male mice (Fig. 2, B and C). In addition, we evaluated the amplitude and latency of wave I of the ABR waveforms, which reflects neural activity in cochlea, SGNs, and the cranial nerve VIII. Mice treated with cisplatin/vehicle exhibited no statistically significant differences in ABR wave I amplitudes (fig. S4, A and B). The latency of wave I was significantly delayed in cisplatin/vehicle-treated mice at 8 and 11.2 kHz in mice cotreated with cisplatin and PLX3397 (fig. S4, C and D). These data show that while wave I amplitudes are less affected, wave I latency was significantly delayed in cisplatin/vehicle-treated animals, and this delay was significantly reduced by ablation of macrophages via PLX3397.

Since cisplatin treatment leads to death of cochlear outer hair cells (OHCs) (*30*), we next measured distortion product otoacoustic emissions (DPOAEs), indirectly measuring OHC function. Mice treated with cisplatin alone (cisplatin/vehicle) demonstrated significant reductions in DPOAE amplitudes at all frequencies (8 to 40 kHz) (Fig. 2, D to I). Mice treated with cisplatin/PLX3397 showed higher DPOAE amplitudes compared to mice treated with cisplatin alone (cisplatin/vehicle), suggesting that macrophage ablation protected against cisplatin-induced OHC dysfunction (Fig. 2, D to I). While macrophage ablation by PLX3397 protected against cisplatin-induced OHC dysfunction in both male and female mice, we observed a sex difference in the protective effect of PLX3397 against cisplatin-induced OHC dysfunction with better protection in male mice (fig. S3).

**Fig. 3.**
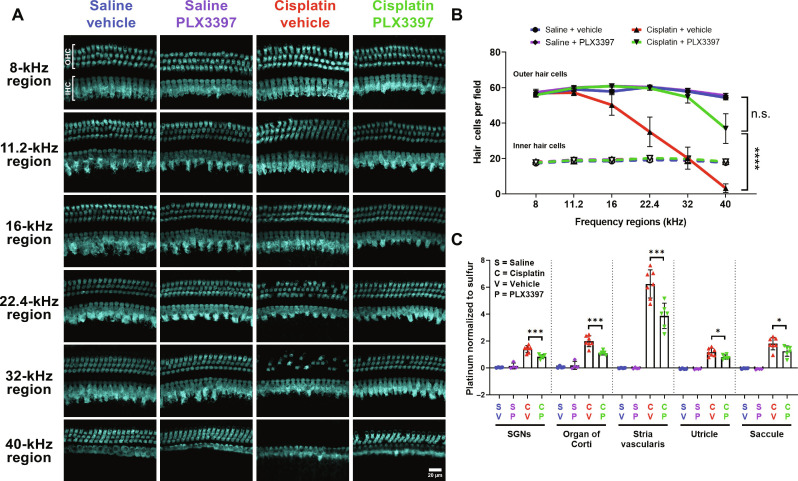
Macrophage ablation followed by partial repopulation via PLX3397 increased OHC survival and decreased cisplatin accumulation in the cochleae of cisplatin-treated mice (experiment 1). (**A**) Microdissected cochlear turns were immunostained for Myosin 7a (cyan) and imaged to assess OHC and IHC across the cochlea. Scale bar, 20 μm. (**B**) Quantification of OHC and IHC numbers. The solid lines (blue, purple, red, and green) represent OHC counts, and dotted lines represent IHC counts. Means ± SEM, *n* = 6 cochleae (3 females and 3 males) per experimental group (total 24 cochleae; 12 females and 12 males). Statistical analysis was performed using two-way ANOVA with Tukey’s multiple comparisons test (main column effect). (**C**) Platinum concentrations in microdissected inner ear tissues were measured using ICP-MS. Means ± SD, *n* = 4 to 7 inner ears (2 to 4 females and 2 to 4 males) per experimental group (total 22 cochleae; 11 females and 11 males). Statistical analysis was performed using one-way ANOVA with Tukey’s multiple comparisons test.

After the end point auditory tests, mice were euthanized, and inner ear tissues were harvested. Cochlear wholemounts were stained for Myosin 7a to visualize inner hair cells (IHCs) and OHCs. Cochleae from control mice (saline/vehicle-treated mice) showed normal cochlear morphology with three rows of OHCs and a single row of IHCs (Fig. 3A). Mice treated with PLX3397 alone did not show any hair cell loss. Cochleae of cisplatin-treated mice showed significant loss of OHCs at cochlear regions corresponding to frequencies of 22.4 kHz and higher. In contrast, mice cotreated with cisplatin and PLX3397 showed greater OHC survival in these high-frequency regions compared to those treated with cisplatin alone (cisplatin/vehicle) (Fig. 3B).

Cisplatin has been shown to cause damage to SGNs (*34*) in addition to hair cells (*30*). To investigate the effects of cisplatin and macrophage ablation by PLX3397 on SGNs, we immunostained cochlear sections for the neuronal marker Tuj-1. Quantitative analyses of three regions of the cochlea (apex, middle, and base) showed that cisplatin resulted in decreased SGN density at the apex, but not in the middle or basal cochlear regions. Cotreatment with cisplatin and PLX3397 resulted in preservation of SGN cell bodies in the apical region of the cochlea (fig. S5). Together, these data indicate that macrophage ablation was protective against cisplatin-induced hearing loss, OHC death, and SGN loss.

Previously, we demonstrated that cisplatin enters the cochlea within 1 hour after systemic administration and is retained indefinitely in both mouse and human cochleae (*7*). To elucidate the underlying mechanisms by which macrophage ablation by PLX3397 confers protection against cisplatin-induced ototoxicity, we examined whether PLX3397 alters cochlear uptake or retention of cisplatin. Platinum levels in microdissected cochlear tissues (SGNs, organ of Corti, and stria vascularis) and vestibular tissues (utricle and saccule) of the inner ear were measured using inductively coupled plasma mass spectrometry (ICP-MS). Consistent with our previous results (*7*), platinum levels were significantly increased in the inner ears of cisplatin-treated mice, with the highest platinum accumulation observed in the stria vascularis (Fig. 3C). Mice cotreated with cisplatin and PLX3397 showed significantly less platinum accumulation in all microdissected inner ear tissues. Notably, the most substantial difference was observed in the stria vascularis, which showed a 33% reduction in cisplatin accumulation in PLX3397-treated animals compared to those treated with cisplatin alone (cisplatin/vehicle) (Fig. 3C). These data indicate that the protective effect of macrophage ablation by PLX3397 is likely related to a reduction in cisplatin accumulation in the inner ear.

### Complete and sustained macrophage ablation fully protects against cisplatin-induced ototoxicity

**Fig. 4.**
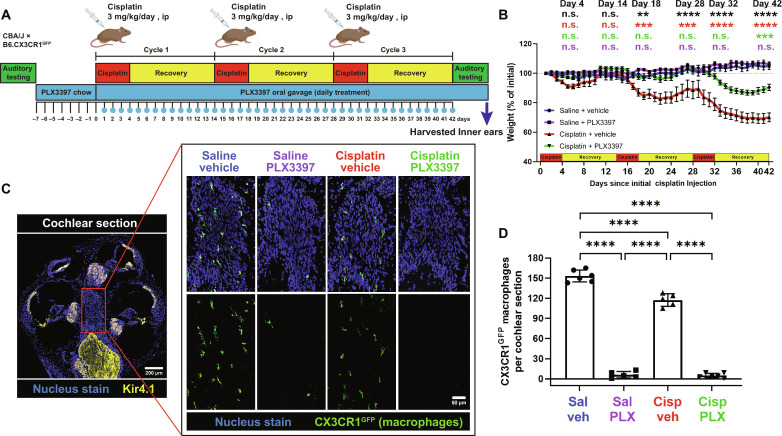
PLX3397 in rodent chow followed by daily oral gavage resulted in sustained macrophage ablation and a significant reduction in weight loss induced by cisplatin (experiment 2). (**A**) Experimental design of experiment 2. Mice received either control chow or PLX3397-formulated chow for 7 days to facilitate macrophage depletion. Following initiation of cisplatin administration, mice received daily PLX3397 treatment via oral gavage, which continued until euthanasia, ensuring sustained macrophage ablation. The days on which mice received PLX3397 through oral gavage are indicated with blue circles. (**B**) Changes in mouse weight are indicated across the 3 cycles of cisplatin administration protocol. Means ± SEM, *n* = 8 to 9 mice per experimental group. Statistical analysis was performed using one-way ANOVA with Tukey’s multiple comparisons test. Statistical comparisons (asterisks or n.s.) are color-coded as described in Methods. (**C** and **D**) Macrophages expressing CX3CR1^GFP^ (green) are visualized in the cochlear modiolus. (C) Scale bars, 200 μm (cochlear section) and 50 μm (modiolus, magnified inset). (D) Quantification of macrophages in whole cochlear sections. Means ± SD, *n* = 5 to 6 cochleae (2 females and 4 males) per experimental group (total 22 cochleae; 8 females and 16 males). Statistical analysis was performed using one-way ANOVA with Tukey’s multiple comparisons test.

In the study presented above and in Figs. 1 to 3 (experiment 1), we showed that macrophage ablation using PLX3397 reduces cisplatin-induced hearing loss and inner ear damage. However, the PLX3397 treatment regimen used in experiment 1 was insufficient to achieve complete macrophage ablation, as demonstrated by the partial macrophage repopulation observed (fig. S1). To address this limitation and to test the effects of more complete macrophage ablation, we repeated the study with a modified experimental design wherein mice were fed with control or PLX3397-formulated chow for 7 days before cisplatin treatment, followed by daily PLX3397 (50 mg/kg) administration via oral gavage throughout the cisplatin administration protocol (experiment 2; Fig. 4A). Mice that received PLX3397 via chow for 7 days followed by daily oral gavage effectively ablated 96% of all CX3CR1^GFP^-positive macrophages within cochlear sections, compared to saline/vehicle-treated mice (Fig. 4, C and D). Macrophage ablation via PLX3397 was consistent across different compartments of the cochlea, including Rosenthal’s canal and modiolus (fig. S6, A, B, and D); as expected, macrophages were not observed in the organ of Corti (fig. S6C) (*13*). Furthermore, we extended our analysis to visualize CX3CR1^GFP^-positive macrophages from cochlear and stria vascularis wholemounts to comprehensively evaluate the extent of macrophage ablation by PLX3397. Quantification of CX3CR1^GFP^-positive macrophages in both tissues revealed an ablation efficiency of >88% in mice administered PLX3397 (fig. S7), without affecting the peripheral immune cells in the spleen, including macrophages, T cells, B cells, natural killer (NK) cells, and neutrophils (fig. S8). Similar to experiment 1, the treatment with cisplatin alone (cisplatin/vehicle) again resulted in a 20 to 25% reduction in cochlear macrophages, specifically in the modiolus, osseous spiral lamina, and the stria vascularis (Fig. 4, C and D, and figs. S6A and S7). Macrophage activation in the cochlea was evaluated by immunofluorescence using Iba-1 antibody. Upon cisplatin treatment, Iba-1 expression level appeared to remain at baseline, as observed in cochleae from control, saline-treated animals, suggesting that macrophages do not become activated during cisplatin treatment (fig. S9, A and B). To further evaluate the infiltration of peripheral immune cells in the cochlea, we visualized CX3CR1^GFP^-positive macrophages that were both positive and negative for CD45 (leukocyte common antigen). The quantification of [CX3CR1^GFP^^+^ CD45^+^] macrophages showed a reduced number of macrophages following cisplatin treatment in the cochlea, consistent with Figs. 1E and 4D. However, the number of [CX3CR1^GFP^^−^ CD45^+^] immune cells revealed no statistically significant differences in cochlear sections, suggesting that peripheral immune cell infiltration into the cochlea was an unlikely event following cisplatin treatment. The percentage of [CX3CR1^GFP^^+^ CD45^+^] immune cells relative to total CD45^+^ cells suggests that more than 95% of all CD45^+^ leukocytes are macrophages in the cochlea of both saline- and cisplatin-treated mice (fig. S9, C to E) (*13*). However, the macrophage infiltration and activation status can vary throughout the cisplatin administration protocol. Therefore, further characterization of macrophage infiltration and activation at intermediate time points during the cisplatin administration protocol, both immediately after cisplatin injections and after recovery period, is required. Together, these data demonstrate that CSF1R inhibition via PLX3397 chow followed by daily oral gavage leads to sustained depletion of resident cochlear macrophages while minimally affecting the peripheral immune cells in both saline- and cisplatin-treated mice.

**Fig. 5.**
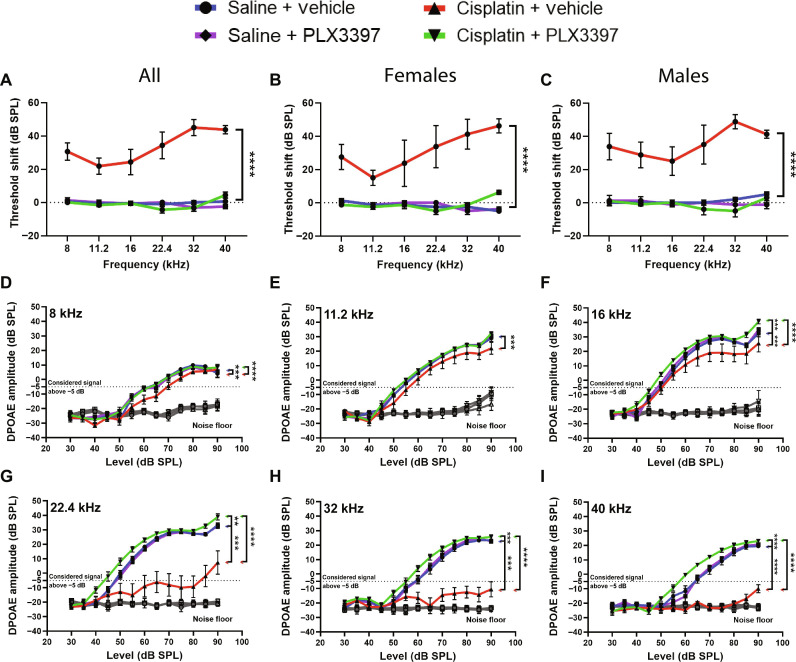
Sustained depletion of macrophages via PLX3397 provided complete protection against cisplatin-induced hearing loss and OHC dysfunction (experiment 2). (**A** to **C**) Hearing loss was assessed by ABRs before (baseline) PLX3397 treatment and after (end point) completion of the cisplatin administration protocol. Hearing loss is reported as threshold shifts (the difference between baseline and end point ABR thresholds). ABR threshold shifts were assessed in both (B) female and (C) male mice. (**D** to **I**) OHC function was evaluated using DPOAEs. An emission at 2*f*_1_ − *f*_2_ was considered present when its amplitude exceeded the threshold of −5 dB (dotted lines). The gray line represents the biological noise floor. For both ABRs and DPOAEs, groups include saline/vehicle-treated mice (blue line), saline/PLX3397-treated mice (purple line), cisplatin/vehicle-treated mice (red line), and cisplatin/PLX3397-treated mice (green line). Data are shown as means ± SEM, *n* = 8 to 9 mice (4 to 5 females and 4 to 5 males) per experimental group (total 34 mice; 16 females and 16 males). Statistical analysis was performed using two-way ANOVA with Tukey’s multiple comparisons test (main column effect).

**Fig. 6.**
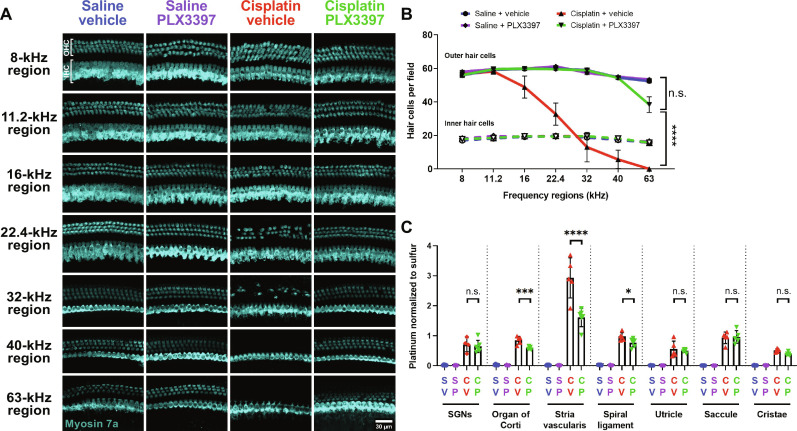
Sustained macrophage ablation protected against cisplatin-induced OHC death and resulted in reduced cisplatin accumulation in the cochlea (experiment 2). (**A** and **B**) Cochlear wholemounts were stained for Myosin 7a (cyan) to visualize hair cells. (A) Representative images and (B) quantitative analysis of Myosin 7a–positive hair cells. Scale bar, 100 μm. (B) Data are shown as means ± SEM, *n* = 6 cochleae (3 females and 3 males) per experimental group (total 24 cochleae; 12 females and 12 males). Statistical analysis was performed using two-way ANOVA with Tukey’s multiple comparisons test (main column effect). (**C**) Platinum levels were measured using ICP-MS in microdissected inner ear tissues. Data are shown as means ± SD, *n* = 4 to 6 cochleae (2 to 3 females and 2 to 3 males) per experimental group (total 22 cochleae; 12 females and 10 males). Statistical analysis was performed using one-way ANOVA with Tukey’s multiple comparisons test.

Throughout experiment 2, mice were weighed daily, and weights are reported as a percentage of initial body weight on day 1. Mice in both saline/vehicle- and saline/PLX3397-treated groups exhibited modest weight gain throughout the study. While all cisplatin-treated mice demonstrated significant weight loss, mice cotreated with cisplatin and PLX3397 exhibited significantly less weight loss compared to those treated with cisplatin alone (cisplatin/vehicle) (Fig. 4B). To determine whether sustained macrophage ablation by PLX3397 protected against cisplatin-induced hearing loss in experiment 2, we again measured ABRs and DPOAEs. Mice treated with cisplatin alone (cisplatin/vehicle) had significant hearing loss (ABR threshold shifts) and substantial reduction in DPOAE amplitudes at all frequencies tested (Fig. 5). Mice coadministered cisplatin and PLX3397 displayed virtually no ABR threshold shifts and complete protection of OHC function, suggesting that PLX3397 treatment provided complete protection against cisplatin-induced hearing loss (Fig. 5A). This complete protection was observed in both male and female animals (Fig. 5, B and C, and fig. S10). In addition, mice treated with cisplatin/vehicle exhibited minimal to no reduction in wave I amplitude but demonstrated significant delays in wave I latencies at 8 and 11.2 kHz. Mice coadministered cisplatin and PLX3397 had wave I latencies that were not different from saline-treated mice at both 8 and 11.2 kHz (fig. S11). Moreover, mice treated with PLX3397 also demonstrated near-total protection against cisplatin-induced OHC death (Fig. 6, A and B). Together, these data indicate that sustained macrophage ablation by PLX3397 provides complete protection against cisplatin-induced hearing loss, OHC dysfunction, and OHC death.

Although cisplatin does not result in the death of IHCs in the cochlea, it does result in the loss of specialized ribbon synapses that mediate rapid and sustained release of neurotransmitters from IHCs (*35*). To assess the effects of cisplatin and macrophage ablation on IHC synapses, we stained cochlear wholemounts for both C-terminal binding protein 2 (CtBP2), which labels presynaptic ribbons, and for GluR2, an AMPA receptor subunit that labels postsynaptic glutamate receptors. Numbers of juxtaposed CtBP2-GluR2 puncta (“synapses”) per IHC and CtBP2-positive puncta (“orphan ribbons”) were quantified. PLX3397 treatment alone did not result in any loss of synapses compared to controls (saline/vehicle-treated mice) (fig. S13, A and B). Mice treated with cisplatin alone (cisplatin/vehicle) demonstrated synaptic loss in the lower-frequency (8, 11.2, and 16 kHz) regions (fig. S13, A and B). Some animals treated with cisplatin/vehicle displayed an increase in the number of orphan ribbons, characterized by the absence of juxtaposed GluR2 in relation to the CtBP2 (fig. S13, A and C) (*36*). Ablation of macrophages using PLX3397 in cisplatin-treated mice resulted in complete protection against loss of synapses induced by cisplatin (fig. S13, A to C). Concurrent with the loss of synapses, quantification of SGN cell bodies revealed a significant decrease in SGN density in the apical cochlear regions in cisplatin-treated mice, while mice that received both cisplatin and PLX3397 exhibited significant protection against cisplatin-induced loss of SGN cell bodies (fig. S13, D and E). These data demonstrate that sustained macrophage ablation using PLX3397 provided complete protection against cisplatin-induced loss of SGNs and concomitant loss of synapses in the inner ear.

We again examined whether administration of PLX3397 reduced cisplatin accumulation in the inner ear in experiment 2 using ICP-MS. Platinum was significantly increased in cochlear and vestibular tissues from cisplatin-treated mice relative to saline-treated mice, with the highest accumulation again observed in the stria vascularis. PLX3397 treatment resulted in reduced platinum levels in the stria vascularis, organ of Corti, and spiral ligament (Fig. 6C). Overall, sustained macrophage ablation in experiment 2 led to an average reduction in platinum accumulation of 45.05% in the stria vascularis, compared to the 33.62% reduction observed in experiment 1 where we observed partial macrophage repopulation. Together, these data suggest a greater reduction in platinum accumulation within the stria vascularis with sustained depletion of macrophages in the cochlea, supporting the idea that the protective effect of macrophage ablation via PLX3397 occurs through a mechanism involving the reduction of cisplatin uptake into the inner ear.

### Macrophage ablation using PLX3397 confers protection against cisplatin-induced nephrotoxicity

**Fig. 7.**
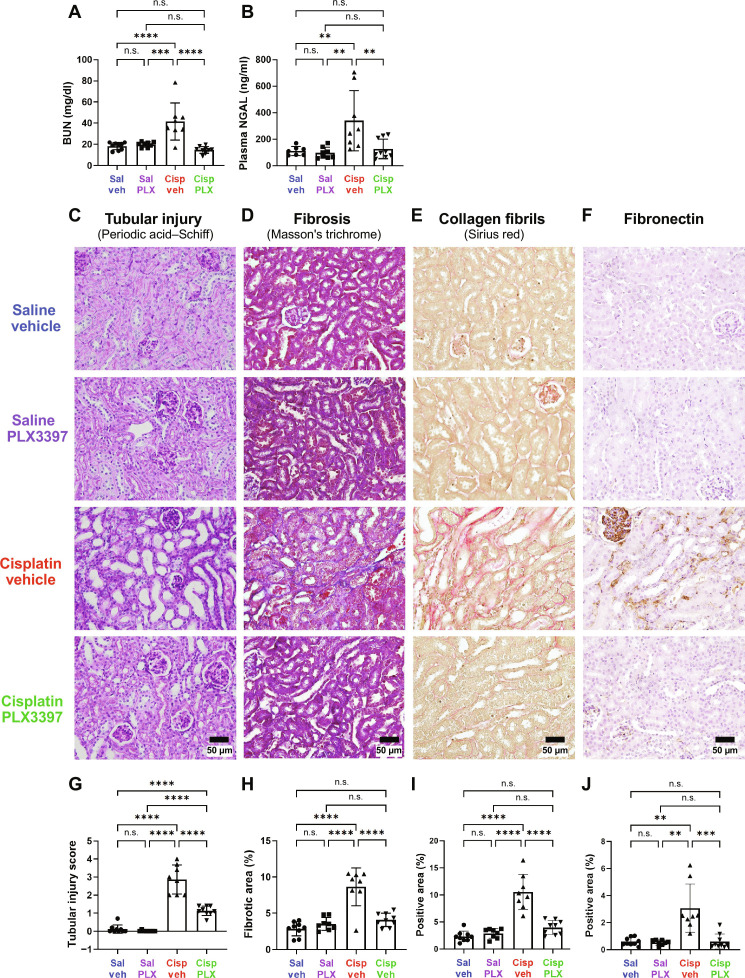
Sustained depletion of macrophages protects against cisplatin-induced kidney damage (experiment 2). Kidney function, tubular injury, and fibrosis were evaluated after end point auditory testing. Macrophage ablation using PLX3397 protected against cisplatin-induced increases in (**A**) plasma BUN levels and (**B**) NGAL levels. (**C** to **F**) Representative images of corticomedullary junction are presented. Scale bars, 50 μm. (C) and (**G**) Tubular injury was semiquantitatively assessed using periodic acid–Schiff staining, and tubular injury scores were assigned (see Methods). (D) and (**H**) Fibrosis was assessed using Masson’s trichrome staining, and the percentage of fibrotic area in the entire surface area of the section was calculated. (E) and (**I**) Fibrillar collagen–positive areas were assessed by Sirius red staining and (F) and (**J**) fibronectin-positive areas shown and evaluated. (G to J) Data are expressed as means ± SD, *n* = 8 to 9 blood and kidney samples (4 females and 4 to 5 males) per experimental group (total 34 mice; 16 females and 18 males). *P* values were calculated using one-way ANOVA with Tukey’s multiple comparisons test.

Cisplatin causes both ototoxicity and nephrotoxicity. Therefore, we evaluated the effects of macrophage ablation using PLX3397 treatment on cisplatin-induced kidney dysfunction and injury (*30*). To examine this, we analyzed blood and kidney tissues. Following the completion of cisplatin administration and auditory tests, biomarkers of kidney function and injury, including plasma blood urea nitrogen (BUN) and neutrophil gelatinase-associated lipocalin (NGAL) levels, were measured from plasma samples obtained from both experiment 1 and experiment 2. Mice treated with cisplatin exhibited significantly elevated levels of plasma BUN and NGAL. However, PLX3397 prevented cisplatin-induced elevation of both BUN and NGAL levels, suggesting that macrophage ablation protected against cisplatin-induced renal dysfunction in both male and female mice (Fig. 7, A and B, and figs. S14, A and B, and S16). Furthermore, histological assessments of kidney tissues obtained from experiment 2, using periodic acid–Schiff, Masson’s trichrome staining, Sirius red staining visualizing higher-order collagen fibrils, and fibronectin staining, revealed a substantial induction of kidney injury and maladaptive repair, characterized by significant tubular injury and interstitial fibrosis, in cisplatin-treated mice (Fig. 7, C to J, and fig. S14, C to F). Macrophage ablation using PLX3397 provided significant protection against both cisplatin-induced tubular injury and interstitial fibrosis in the kidney (Fig. 7, C to J). The protective effect of PLX3397 was observed in both female and male mice treated with cisplatin (fig. S14). In addition, previous reports have indicated that cisplatin-induced nephrotoxicity is characterized by activation of inflammatory cytokines (*37*, *38*). To investigate the effect of PLX3397 on cisplatin-induced inflammation, we evaluated the gene expression of cytokines (*Tnf* and *Tgfb1*) and Toll-like receptors (*Tlr2* and *Tlr4*) in kidney tissues. Transforming growth factor–β (TGF-β; the protein encoded by *Tgfb1* gene) is also the key mediator of renal fibrosis. Our data indicate a significant increase in the gene expression of inflammatory cytokines and Toll-like receptors, following cisplatin treatment. PLX3397 treatment led to a significant reduction in the levels of cytokines (*Tnf* and *Tgfb1*) and Toll-like receptors (*Tlr2* and *Tlr4*), suggesting that the inflammatory profile in the kidney is reduced with PLX3397 treatment (fig. S15). Together, these data indicate that PLX3397 effectively (i) protected against cisplatin-induced renal tissue damage, (ii) mitigated associated pathological changes, and (iii) reduced inflammatory responses.

**Fig. 8.**
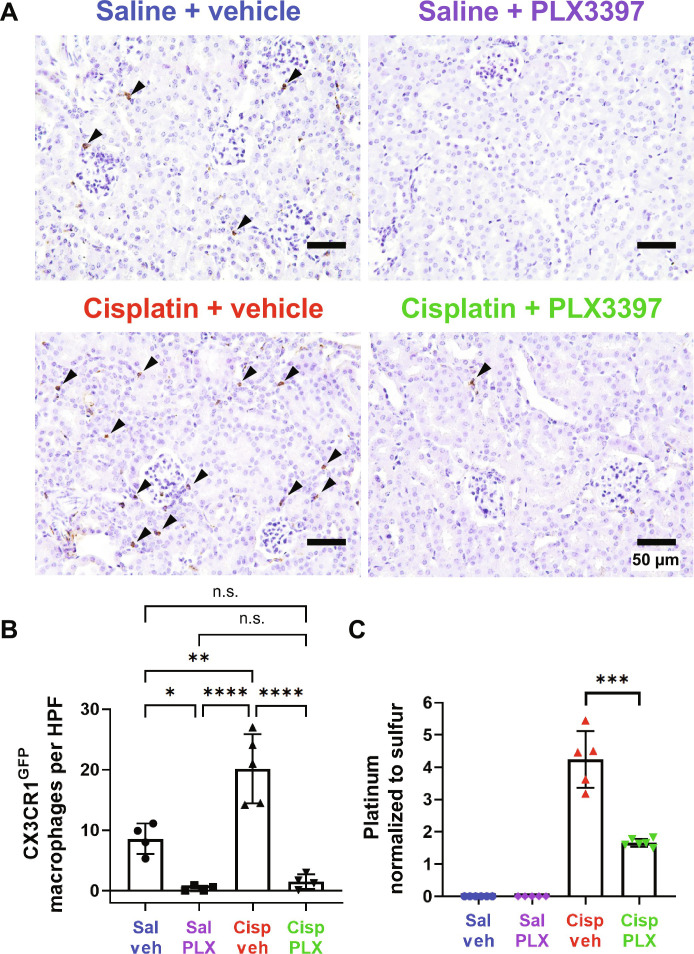
PLX3397 results in ablation of CX3CR1^GFP^-positive cells and significant reduction in cisplatin accumulation in the kidney (experiment 2). (**A**) Representative images of renal CX3CR1^GFP^-positive cells (arrowheads), identified through immunohistochemical staining. Scale bars, 50 μm. (**B**) The number of CX3CR1^GFP^-positive cells in each field was quantified. Data are expressed as means ± SD, *n* = 4 to 5 kidney samples (2 females and 2 to 3 males) per experimental group (total 17 mice; 8 females and 9 males). Compared to control mice (saline/vehicle-treated mice), PLX3397 treatment in saline-treated mice and cisplatin-treated mice ablated 95.63 and 82.52% of CX3CR1^GFP^-positive cells, respectively. HPF, high-power field (the area of a slide visible under ×400 magnification of corticomedullary junction). (**C**) Platinum levels in the kidney tissues were analyzed by ICP-MS. Data are expressed as means ± SD, *n* = 5 to 6 blood and kidney samples (2 to 3 females and 2 to 3 males) per experimental group (total 22 mice; 11 females and 11 males). Cisplatin resulted in increased platinum in the kidney, while PLX3397 significantly reduced cisplatin accumulation. Platinum levels were normalized to sulfur contained in the kidney. Data are expressed as means ± SE. *P* values were calculated using one-way ANOVA with Tukey’s multiple comparisons test.

Administration of PLX3397 has been demonstrated to ablate microglia (*31*) and cochlear macrophages (Figs. 1 and 4 and fig. S7). However, the effects of PLX3397 treatment on kidney-resident macrophages had not been evaluated. Therefore, we assessed the impact of PLX3397 on kidney-resident macrophages. We observed a significant (2.36-fold) increase in the number of CX3CR1^GFP^-positive cells within the kidneys of cisplatin/vehicle-treated mice compared to the kidneys of control mice treated with saline/vehicle (Fig. 8, A and B). Consistent with our observations in the cochlea, PLX3397 resulted in a marked decrease of 95.6% in saline-treated mice (saline/vehicle versus saline/PLX3397) and a reduction of 92.6% in cisplatin-treated mice (cisplatin/vehicle versus cisplatin/PLX3397) of CX3CR1^GFP^-positive cells, primarily macrophages, in the kidney (Fig. 8, A and B).

To begin to examine the mechanisms underlying the protective effects of PLX3397 against cisplatin-induced nephrotoxicity, we investigated whether PLX3397 reduced cisplatin accumulation in the kidney. Platinum levels in kidney tissues were analyzed using ICP-MS. As expected, platinum levels were significantly elevated (4.24-fold) in kidneys from cisplatin/vehicle-treated mice relative to those from saline/vehicle-treated mice. Kidneys from mice cotreated with cisplatin and PLX3397 contained 60.88% less platinum compared to the kidneys of mice treated with cisplatin and vehicle (Fig. 8C). These data indicate that macrophage ablation by PLX3397 protected against cisplatin-induced nephrotoxicity as assessed by biomarkers of kidney function, tubular injury, and interstitial fibrosis, and they suggest that this protective effect is mediated through a mechanism involving reduced cisplatin uptake and/or accumulation in the kidney.

## DISCUSSION

Cochlear macrophages play an important role in homeostasis and during cochlear injury. In noise damage models that result in the loss of hair cell synaptic ribbons, macrophages are involved in synaptic repair and the restoration of cochlear synapses and synaptic function (*12*). Here, we show that macrophages also play important roles in the cochlear and kidney responses to cisplatin-induced damage, in part by regulating cisplatin uptake and accumulation in the cochlea and kidney.

### Macrophage depletion and cisplatin-induced hearing loss

Our experimental design of macrophage ablation in a mouse model of cisplatin ototoxicity used two methods to administer PLX3397. In both experiment 1 and experiment 2, the use of PLX3397-formulated chow for 7 days resulted in the ablation of >88% of all cochlear macrophages. However, we observed some macrophage repopulation in experiment 1, which was ameliorated by increasing the frequency of PLX3397 administration. Data from both studies consistently demonstrated that macrophage ablation conferred significant protection against cisplatin-induced hearing loss, OHC dysfunction and death, loss of SGNs, and a concomitant loss of synapses in the inner ear. We have elected to present both studies herein because they collectively demonstrate that the extent of macrophage ablation affects the magnitude of the protective effect. Notably, experiment 2, characterized by sustained macrophage ablation, exhibited considerably higher levels of protection, specifically in female mice, approaching near-complete preservation of auditory function and hair cells compared to experiment 1, in which macrophage repopulation occurred during the cisplatin administration protocol (fig. S12).

The major route of cisplatin entry into the cochlea is thought to be through the BLB (*39*). The BLB includes the blood-strial barrier and the blood-perilymph barrier (*40*). The blood-strial barrier is situated between the blood flow and the intrastrial space located between the marginal cells and intermediate cells within the stria vascularis, which contains a dense network of strial capillaries, and is known to be the primary route of cisplatin entry. The blood-perilymph barrier separates blood from perilymph that fills the scala vestibuli and scala tympani and is found in the vasculature located in the modiolus, spiral limbus, SGN, and osseous spiral lamina (*41*, *42*). The BLB is composed of vascular endothelial cells connected to each other by tight junctions surrounded by pericytes and PVMs. PVMs are closely associated with capillaries and regulate the BLB permeability (*15*, *16*). PVMs are primarily found in the stria vascularis but are also present in other capillary regions, including the vasculature in the modiolus, and they are closely associated with both the blood-strial and blood-perilymph barriers. Therefore, while the blood-strial barrier in the stria vascularis is thought to be the primary route of cisplatin into the cochlea (*39*), cisplatin may enter the cochlea through the blood-perilymph barrier, and it would be beneficial for future studies to investigate additional routes of cisplatin entry into the inner ear. This investigation could help identify additional targets for therapeutic drugs to prevent cisplatin-induced hearing loss or strategies to regulate blood-perilymph barrier, permeability to further exclude cisplatin from the cochlea.

**Fig. 9.**
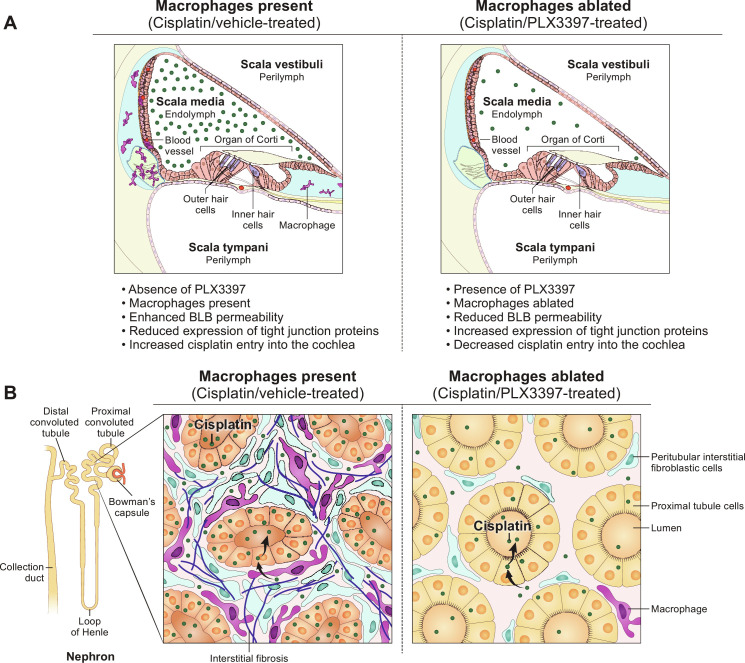
Working model of the mechanisms underlying the protective effects of macrophage ablation against cisplatin-induced hearing loss and kidney injury. (**A**) In the absence of PLX3397, macrophages are present, and the cochlear BLB is permeable enough to permit cisplatin to cross the BLB and enter the cochlea, where it results in the death of OHCs, loss of ribbon synapses and SGNs, and hearing loss. In the presence of PLX3397, PVMs are ablated, and BLB permeability is reduced, resulting in reduced cisplatin entry and protection against cisplatin-induced hearing loss. (**B**) In the absence of PLX3397, renal macrophages are present and may promote cisplatin entry into proximal tubule cells causing renal fibrosis and kidney dysfunction. In the presence of PLX3397, macrophages are ablated in the kidney, and cisplatin accumulation in the kidney is reduced, thus providing protection against cisplatin-induced tubular injury and interstitial fibrosis.

Treatment with PLX3397 resulted in ablation of PVMs within the stria vascularis (fig. S7, C and D) and reduced platinum levels in the cochlea, with the highest reduction observed in the stria vascularis (Figs. 3C and 6C). Thus, our data are consistent with a model in which ablation of PVMs in the stria vascularis resulted in reduced permeability of the BLB and consequently decreased cisplatin entry into the cochlea. The precise cellular and molecular mechanisms by which PVMs regulate BLB permeability during cisplatin treatment remain largely unknown and are likely to involve multiple factors. One proposed mechanism by which the permeability of the BLB may be regulated involves the interaction between PVMs and vascular endothelial cells. PVMs can secrete soluble factors that subsequently bind to their receptors expressed on vascular endothelial cells lining the capillary lumen. This interaction can modulate the expression levels of tight junction proteins in the endothelial cells, thereby influencing BLB permeability (*15*, *18*). Therefore, in the context of cisplatin treatment, we propose that soluble factors produced by PVMs may bind to their receptors expressed on vascular endothelial cells, subsequently down-regulating tight junction proteins connecting the vascular endothelial cells. This can result in increased permeability of the BLB (leaky BLB), thereby facilitating the entry of cisplatin into the inner ear (Fig. 9A). In the absence of PVMs, the soluble factors responsible for BLB breakdown are absent, BLB integrity is maintained, and cisplatin entry into the inner ear is reduced. This model is consistent with a recent report demonstrating that cisplatin-induced hearing loss was associated with increased BLB permeability and reduced expression of tight junction proteins between vascular endothelial cells (*43*). This hypothesis also provides a plausible explanation for the differences in the extent of protection against cisplatin-induced hearing loss that we observed between experiment 1 and experiment 2. Our data are consistent with a model in which macrophage repopulation in experiment 1 may have allowed for the release of soluble factors that disrupted the integrity of the BLB, increasing its permeability and thus increasing the entry of cisplatin into the cochlea. In experiment 2, sustained ablation of macrophages ensured the maintenance of BLB integrity, thereby limiting the entry of cisplatin into the cochlea. We observed a significant reduction in cochlear platinum levels following macrophage ablation by PLX3397 in both experiment 1 and experiment 2, with the largest reduction in the stria vascularis. In experiment 1, in which partial macrophage repopulation was observed, platinum accumulation in the stria vascularis was reduced by 33.62% in PLX3397-treated animals. In experiment 2, there was sustained macrophage ablation, and we observed a larger 45.04% decrease in cisplatin accumulation (Figs. 3C and 6C). These data suggest that macrophage repopulation in experiment 1 was associated with greater cisplatin uptake and accumulation in the cochlea compared to experiment 2, in which macrophage ablation was sustained.

Macrophage ablation via PLX3397 partially reduced cisplatin accumulation in the cochlea, while the protection mediated by PLX3397 against cisplatin-induced ototoxicity was near-complete. Two potential explanations may account for this observation. First, there may be a “threshold” concentratioxn for cisplatin to cause ototoxicity. Damage to hair cells may only occur when the concentration of cisplatin exceeds this threshold and is exposed for certain length of time. Previously, we have shown that animals subjected to hearing tests after the first cycle of cisplatin injection (4 days of cisplatin treatment) and the subsequent recovery period (4 days of cisplatin treatment followed by 10-day recovery period) exhibited no ABR threshold shift, indicating normal hearing (*7*). However, during cycle 1 period, a considerable amount of platinum was still present in the cochlea, suggesting that the near-complete protection observed in the current study may be attributed to only a partial reduction in cisplatin accumulation within the cochlea (*7*). Second, although the decrease in platinum levels in the cochlea following macrophage ablation suggests a role for macrophages in regulating cisplatin entry into the cochlea, this may not fully account for the substantial protection observed against cisplatin-induced ototoxicity. Here, we have observed that while macrophage ablation via PLX3397 resulted in the most substantial reduction in platinum levels within the stria vascularis, our analyses of other microdissected inner ear tissues (SGNs, organ of Corti, spiral ligament, utricle, saccule, and cristae) revealed minimal differences in platinum levels in both studies following macrophage ablation in cisplatin-treated mice, in comparison to mice with intact macrophages (Figs. 3C and 6C). Thus, additional macrophage-dependent factors may contribute to cisplatin-induced ototoxicity. Cisplatin ototoxicity is associated with increased production of reactive oxygen species generated through NADPH (reduced form of nicotinamide adenine dinucleotide phosphate) oxidase 3 (*44*, *45*) and secretion of proinflammatory cytokines such as tumor necrosis factor–α (TNFα), interleukin-1β (IL-1β), and interleukin-6 (IL-6) (*46*) through activation of nuclear factor κB (NF-κB), a transcription factor and regulator of innate immunity (*47*). Both oxidative stress and proinflammatory cytokines exert cytotoxic effects in the context of cisplatin-induced ototoxicity (*48*, *49*). It is plausible that macrophages may also contribute to the production of these cytotoxic factors. Emerging technologies such as single-cell resolution spatial transcriptomics may provide insight into the functional state of macrophages in response to cisplatin exposure in the inner ear.

Upon entering the stria vascularis from the BLB, cisplatin subsequently enters the endolymph within the cochlear duct (*50*, *51*). Once inside the cochlea, cisplatin can enter various cell types, including hair cells, SGNs, and stria vascularis, via the mammalian cation transporters or possibly via mechanoelectrical transduction channels (*52*, *53*) and can exert cytotoxic effects at the level of individual cells. Following systemic administration, cisplatin is present in the organ of Corti, SGNs, stria vascularis, spiral ligament, utricle, saccule, and cristae (Figs. 3C and 6C) (*7*). Upon entry into the OHCs, cisplatin irreversibly binds to cellular macromolecules (DNA, RNA, and proteins) and leads to apoptotic degeneration of OHCs and permanent hearing loss (*1*). In contrast to OHCs, IHCs are relatively resistant to the cytotoxic effect of cisplatin, as they appear morphologically intact after cisplatin treatment (Figs. 3, A and B, and 6, A and B) (*30*). However, our data demonstrate a significant loss of IHC synaptic ribbons (fig. S13, A to C) and a reduction in SGN density (figs. S5 and S13, D and E) following cisplatin treatment. Our data revealed a selective loss of IHC synapses in the low-frequency, apical regions of the cochlea upon cisplatin treatment, while the degeneration of OHCs primarily occurred in the high-frequency, basal region of the cochlea. This loss of IHC synapses in the apical region of the cochlea is congruent with the loss of SGN density in the apical regions, while the basal region remained unaffected (figs. S5 and S13, D and E). These observations demonstrate that different cell types within the inner ear exhibit distinct susceptibility profiles in response to cisplatin or other cochlear insults, possibly influenced by tonotopic regions of the organ of Corti or specific organs within the inner ear. For example, basal OHCs are more vulnerable to a variety of cochlear insults, including cisplatin (*30*), gentamicin (*54*), and neomycin (*55*) compared to apical OHCs. Macrophage ablation by PLX3397 treatment in both experiment 1 and experiment 2 resulted in significant protection against cisplatin-induced loss of IHC synaptic ribbons and SGN cell bodies (figs. S5 and S13). These data provide additional evidence in support of the hypothesis that the presence of PVMs increases BLB permeability, leading to increased cisplatin entry into the cochlea, while macrophage ablation maintains BLB integrity, thereby reducing entry of cisplatin into the cochlea and offering protection against cisplatin-induced cellular damage in the inner ear. Thus, our data suggest a crucial role for PVMs in modulating BLB permeability, highlighting their potential as therapeutic targets for mitigating cisplatin ototoxicity.

Previous studies have implicated macrophages in the context of cisplatin-induced hair cell loss. Lee *et al.* (*56*) reported increased inflammatory responses, macrophage recruitment, and phagocytosis of hair cells in zebrafish larvae treated with cisplatin. However, selective depletion of macrophages did not affect the loss of hair cells in this model. In addition, Baker *et al.* (*57*) reported that induction of heme oxygenase-1 (HO-1) in resident macrophages, achieved by treatment with cobalt protoporphyrin IX (CoPPIX), provided significant protection against cisplatin-induced hair cell death in utricle cultures (one of the vestibular organs in the inner ear). Furthermore, the depletion of macrophages from utricles abolished the protective effect of HO-1 induction (*57*), suggesting that macrophages contributed to protection of hair cells against cisplatin-induced damage. In both of these studies, the cisplatin administration method allowed for direct access of cisplatin to sensory hair cells. In contrast, our study used systemic administration of cisplatin, which requires that cisplatin crosses the BLB to enter the cochlea and access hair cells. Our data indicate that macrophage ablation provides significant protection against cisplatin-induced ototoxicity, suggesting a pathogenic role for macrophages in the development of hearing loss and hair cell death. Since the primary difference between zebrafish lateral line and utricle culture compared to the in vivo mouse model used in this study is the absence (or presence) of the BLB and the associated PVMs, our data indicate that in the context of an intact BLB, macrophages regulate the entry of cisplatin into the cochlea and, thus, cochlear hair cells are damaged by cisplatin. The differences between these models may be useful in identifying the distinct roles of resident macrophages versus PVMs on hair cells during cisplatin treatment.

### Depletion of CX3CR1-positive cells and cisplatin-induced nephrotoxicity

Nephrotoxicity is another notable and commonly observed side effect of cisplatin. Therefore, we examined the effects of CSF1R inhibition on cisplatin-induced nephrotoxicity. We began by examining the nephrotoxic effects of cisplatin in our mouse model, which had been developed for ototoxicity (*30*). Consistent with observations in other mouse models of cisplatin-induced nephrotoxicity (*58*, *59*), our 3-cycle cisplatin administration protocol resulted in elevated plasma levels of BUN and NGAL, indicating kidney dysfunction and injury (Fig. 7, A and B, and figs. S14, A and B, and S16). Histological analyses revealed significant tubular injury and interstitial fibrosis in the kidneys of cisplatin-treated mice (Fig. 7, C to J, and fig. S14, C to F), validating the utility of the 3-cycle cisplatin administration protocol as a model for cisplatin-induced nephrotoxicity. We observed that PLX3397 treatment, which primarily ablates macrophages in the kidney, provided significant protection against cisplatin-induced kidney dysfunction and prevented both tubular injury and interstitial fibrosis (Fig. 7, C to J, and fig. S14, C to F). A pathogenic role of macrophages has been described in various models of kidney disease through studies using macrophage depletion. For example, in the genetic ablation model using CD11b–diphtheria toxin receptor (DTR) mice or treatment with liposomal clodronate, macrophage depletion resulted in improved renal function and mitigated pathological damage in response to acute ischemic kidney injury (*60*, *61*) and unilateral ureteral obstruction (*62*). In addition, depletion of kidney-resident macrophages attenuated cisplatin-induced kidney fibrosis in mice that were treated with cisplatin (9 mg/kg) weekly and euthanized 3 days after the last dose (*29*, *63*). In our study, we demonstrated the pathogenic role of CX3CR1-positive cells, primarily macrophages, in our clinically relevant mouse model of cisplatin-induced kidney injury (nephrotoxicity). Furthermore, we elucidated the underlying mechanism by which ablation of CX3CR1-positive cells confers protection against cisplatin-induced nephrotoxicity. This was evidenced by increased levels of platinum observed in the kidneys of cisplatin/vehicle-treated mice, whereas the kidneys of cisplatin/PLX3397-treated mice exhibited significantly less platinum accumulation (Fig. 8C). These data suggest that ablation of CX3CR1-positive cells, primarily macrophages, restricted accumulation of cisplatin in the kidney, thereby offering renal protection by minimizing cisplatin exposure (Fig. 9B).

In addition, inflammatory cytokines and Toll-like receptors are important mediators of cisplatin-induced nephrotoxicity (*37*, *38*). We evaluated the gene expression of cytokines (*Tnf* and *Tgfb1*) and Toll-like receptors (*Tlr2* and *Tlr4*) in kidney tissues. TGF-β (the protein encoded by *Tgfb1* gene) is also recognized as the primary mediator of renal fibrosis. PLX3397 significantly reduced the expression levels of these inflammatory response–related genes (fig. S15). Previous studies indicate that blocking (TNFα) ameliorates cisplatin-induced nephrotoxicity (*37*). These data and ours are consistent with a model in which the protective effect of PLX3397 against cisplatin-induced nephrotoxicity is mediated by prevention of induction of these cytokines and Toll-like receptors through ablation of CX3CR1-positive cells (primarily macrophages).

Another possible mechanism for this protection is that macrophages may regulate the expression or function of transporter proteins that mediate uptake or efflux of cisplatin in proximal tubular cells. Renal epithelial cells take up cisplatin through specific transporters, including organic cation transporter 2 (OCT2) and multidrug and toxin extrusion 1 (MATE1). OCT2 is predominantly expressed in the basolateral membrane of proximal tubular cells and mediates cellular uptake of cisplatin (*64*, *65*), while MATE1 is expressed in the apical brush-border membrane of renal proximal tubular cells and facilitates cisplatin efflux (*66*). It is plausible that macrophages may regulate expression of these cisplatin transporters in proximal tubular cells in a manner that promotes cisplatin entry. In this model, depletion of macrophages would result in reduced expression of OCT2 (restricting entry of cisplatin into the tubular cells) and/or increased expression of MATE1 (enhancing cisplatin efflux) by the tubular cells, ultimately leading to reduced cisplatin accumulation in the kidney.

### Impact of cisplatin and PLX3397 on resident macrophage populations

In our study, cisplatin had an impact on the numbers of macrophages in the cochlea. The administration of cisplatin alone resulted in significant hearing loss and a notable reduction of ~20 to 40% of cochlear macrophages, including PVMs, in both experiment 1 and experiment 2 (Figs. 1, D and E, and 4, C and D; and fig. S7). This observation suggests that cisplatin has induced cell death in a subset of these macrophages. The level of macrophage ablation achieved by PLX3397 treatment was similar in both studies, with >88% of all macrophages eliminated (Figs. 1, D and E, and 4, C and D), and this macrophage ablation resulted in significant protection against cisplatin-induced hearing loss. This suggests that the macrophages that remain after cisplatin treatment contributed to increased cisplatin entry into the cochlea and/or played additional pathogenic roles in response to cisplatin treatment.

We observed a significant increase in the number of CX3CR1^GFP^-positive cells in the kidney at the end of the cisplatin treatment (Fig. 8, A and B). This was in contrast to the decrease in CX3CR1^GFP^-positive cells that we observed in the cochlea of cisplatin-treated animals (Figs. 1, D and E, and 4, C and D). This finding is consistent with previous reports suggesting that peripheral immune cells readily infiltrate the kidney following cisplatin treatment (*59*, *67*). Therefore, this difference between the cochlea and kidney may be attributable to peripheral immune cell infiltration into the kidney. Although CX3CR1 is more commonly expressed in macrophages, CX3CR1 expression has also been observed in circulating monocytes, dendritic cells, NK cells, and T cells (*68*). Therefore, the observed increase in the CX3CR1^GFP^-positive cells within the kidney during cisplatin treatment may indicate infiltration of a heterogeneous population of peripheral immune cells (including macrophages) expressing CX3CR1.

To evaluate the roles of macrophages in cisplatin-induced hearing loss, we have used pharmacological CSF1R inhibition to ablate cochlear macrophages. While several conditional transgenic mouse models of macrophage depletion have been established, the two CSF1R inhibitors, PLX3397 and PLX5622, developed by Plexxikon Inc. (San Francisco, CA), provide various advantages that offer a noninvasive oral administration route, either by chow formulation (*12*, *32*) or oral gavage (*69*). In addition, they enable long-term sustained macrophage ablation (*31*) without inducing an inflammatory response, allowing their use for experiments with long durations and adding a favorable safety profile, as described in this study. However, while pharmacological CSF1R inhibition has demonstrated efficacy and versatility, these small-molecule inhibitors rely on peripheral administration, which depletes not only macrophages in the cochlea but also microglia in the CNS. Therefore, it is plausible that the observed results may not be only attributed to the elimination of cochlear macrophages but also involve the ablation of microglia. As more refined animal models and tools emerge, further investigation to pursue a more targeted approach to depleting cochlear macrophages or PVMs to specifically elucidate their roles in cisplatin-induced hearing loss is warranted.

PLX3397 and PLX5622 were initially thought to have minimal impact on peripheral immune cells. However, recent research has demonstrated that both compounds notably affect tissue-resident macrophages in peripheral organs, such as the peritoneum, lung, and liver (*70*). However, to date, there have been only few reports investigating the impact of CSF1R inhibitors on resident macrophages in the kidney. We observed that sustained macrophage ablation by PLX3397 resulted in a significant reduction in the number of CX3CR1^GFP^-positive cells in the kidney (Fig. 8, A and B). In contrast to its effect on the kidney, PLX3397 had minimal impacts on the populations of leukocytes in the spleen, as assessed by flow cytometry (fig. S8). This is consistent with previous reports indicating that PLX3397 has minimal impact on the population of leukocytes in the spleen, as well as other hematopoietic organs, such as bone marrow or blood (*12*, *33*).

In contrast to the minimal impact PLX3397 had on immune cell populations in the spleen, treatment with cisplatin did result in a significant reduction in the number of immune cells within the spleen (fig. S8). This is consistent with the well-established myelosuppressive effect of cisplatin, which is characterized by reduced bone marrow activity leading to decreased production of red blood cells (RBCs), white blood cells (immune cells), and platelets. Consequently, cisplatin-induced myelosuppression can lead to hematological toxicities, including anemia, leukopenia, neutropenia, and thrombocytopenia (*71*, *72*). Notably, cotreatment of mice with cisplatin and PLX3397 in our study did not result in increased numbers of immune cells in the spleen, suggesting that PLX3397 was not protective against cisplatin-induced myelosuppression (fig. S8).

In summary, our data demonstrate substantial depletion (>88%) of CX3CR1^GFP^-positive macrophages in both the inner ear and the kidney using the CSF1R inhibitor, PLX3397. Macrophage ablation resulted in protection against cisplatin-induced ototoxicity and nephrotoxicity. Specifically, macrophage ablation preserved hearing function and prevented the loss of OHCs, IHC synapses, and SGNs. In the kidney, we observed substantial protection against cisplatin-induced tubular injury and fibrosis and preservation of kidney function. In addition, macrophage ablation resulted in a significant reduction in platinum accumulation in both the inner ear and the kidney. These data suggest that macrophage ablation confers protection against cisplatin-induced ototoxicity and nephrotoxicity by limiting accumulation of cisplatin in the inner ear and kidney. Therefore, our results are consistent with a model in which macrophages play a role in facilitating cisplatin-induced cytotoxicity by promoting the entry of cisplatin into these organs. Since PLX3397 is U.S. Food and Drug Administration (FDA)–approved to treat tenosynovial giant cell tumors, our data suggest a potential therapeutic approach using PLX3397 to prevent the two most commonly occurring toxicities in patients with cancer undergoing cisplatin treatment. A prospective clinical study of PLX3397 that aims to reduce cisplatin-associated ototoxicity and nephrotoxicity in patients with cancer is warranted.

## METHODS

### Animals

Studies were performed using adult mice of F1 offspring from CX3CR1^GFP/GFP^ (C57BL/6 background, stock #005582, the Jackson Laboratory) cross bred with CBA/J (stock #000656, the Jackson Laboratory). C57BL/6 wild-type mice develop sensorineural hearing loss as early as 3 to 4 months of age, much earlier than other mouse strains such as CBA/J (*73*). Therefore, this mouse breeding strategy was used to overcome the early onset hearing loss inherent in the C57BL/6 wild-type mouse strain while maintaining enhanced green fluorescent protein (EGFP) expression in macrophages. CX3CR1^GFP/GFP^ (C57BL/6 background) homozygous mice express EGFP under the control of the endogenous *Cx3cr1* locus, where the *Cx3cr1* gene is replaced with *EGFP* resulting in mice that function as CX3CR1 knockout mice (*74*). Here, we used a CX3CR1^GFP/+^ heterozygous mice in which one copy of the *Cx3cr1* gene is replaced with *EGFP*, while the other copy is still functional. This allows us to maintain the function of CX3CR1 in these mice while also visualizing CX3CR1-expressing cells through the expression of EGFP. Heterozygous mice were genotyped by polymerase chain reaction (PCR) amplification of genomic DNA extracted from tail snips as recommended by the Jackson Laboratory (www.jax.org/jax-mice-and-services/customer-support/technical-support/genotyping-resources/genotyping-protocol-database). The F1 mouse strain with EGFP-labeled macrophages is referred to as CBAJ/CX3CR1^GFP/+^.

### Study design

The study was performed under National Institutes of Health (NIH) National Institute on Deafness and Other Communication Disorders (NIDCD) and National Institute of Neurological Disorders and Stroke (NINDS) Animal Care and Use Committee-approved protocol (#1327). Male and female CBAJ/CX3CR1^GFP/+^ mice (9 to 14 weeks old) were housed individually and assigned to one of four treatment groups: (i) saline/vehicle, (ii) saline/PLX3397, (iii) cisplatin/vehicle, or (iv) cisplatin/PLX3397. Mice were given ad libitum access to food and water. Cisplatin-treated mice received 3 cycles of once-daily cisplatin [3 mg/kg, intraperitoneally (ip)] for 4 days, followed by a 10-day recovery period (3 mg/kg × 4 days × 3 cycles = 36 mg/kg cumulative dose) as described previously (*30*). Control animals received same volume of 0.9% sterile saline. Cisplatin-treated mice received supportive care: (i) daily 1 ml of saline subcutaneous injections in the morning and normasol (Hospira Inc., San Clemente, CA, USA) in the afternoon; (ii) 0.3 ml of signal transducers and activators of transcription high-calorie liquid supplement (PRN Pharmacal, Pensacola, FL, USA) twice daily to maintain body weight. Mice were monitored twice daily for changes in overall health or activity using a daily body condition score, for overall condition (muscular tone, body fat content, coat maintenance, and overall energy level) (*75*).

To achieve experimental macrophage depletion, PLX3397 was administered via a chow formulation, followed by oral gavage. To prepare PLX3397 chow, PLX3397 was purchased from MedChemExpress Inc. (HY-16749, Monmouth Junction, NJ, USA) and sent to Research Diets Inc. (New Brunswick, NJ, USA) to formulate in AIN-76A standard chow at 660 parts per million (ppm) (660 mg/kg). Chows were not irradiated. On average, mice consume ~4 g of chow per day, resulting in a daily intake of 2.64 mg of PLX3397. Standard AIN-76A rodent chow without PLX3397 (D10001, Research Diets Inc.) was purchased to serve as a control chow.

To prepare PLX3397 solution (50 mg/kg) for oral gavage, PLX3397 was dissolved in 100% PEG300 (202371, Sigma-Aldrich) to make a concentration of 25 mg/ml and incubated on a rotating platform at room temperature for 15 to 30 min. Subsequently, this mixture was subjected to sonication in an ultrasonic bath (Branson 1510) for 20 to 40 min to ensure complete solubilization of PLX3397 and stored at −20°C until use. Separately, 10% Tween 80 stock was prepared in saline (0.9% NaCl) and stored at room temperature until use. On the day of treatment, PLX3397/PEG300 solution (25 mg/ml) was mixed with 10% Tween 80/saline to make a final concentration of PLX3397 (10 mg/ml) in 40% PEG300, 4% Tween 80, and 56% saline diluent. Animals assigned to vehicle treatment were given comparable volumes of the diluent without PLX3397. Although dimethyl sulfoxide (DMSO) is commonly used as a component of the solvent to dissolve PLX3397 for oral gavage, it was excluded from our study because of its known ototoxic properties (*76*, *77*). Mice receiving a dose of PLX3397 (50 mg/kg) via oral gavage received an estimated dose of 1.25 mg of PLX3397 per oral gavage for an average mouse weighing 25 g, which is equivalent to a concentration of PLX3397 (~310 mg/kg) in the formulated chow.

Two independent experiments were performed. In both experiments, auditory tests were performed before the start (baseline) and at the end (end point) of the cisplatin administration protocol. Following baseline auditory tests, chow containing 660 ppm of PLX3397 (or control AIN-76A rodent chow) replaced the regular mouse diet for 7 days to ablate (or maintain) macrophages. Following the 7-day pretreatment, mice were returned to the regular rodent chow (5L8F) and underwent the cisplatin administration protocol as they continued to receive PLX3397 via oral gavage. Mice receiving cisplatin demonstrate reduced appetite; therefore, PLX3397 chow feeding could have resulted in insufficient drug administration. Thus, vehicle or PLX3397 (50 mg/kg) was administered via oral gavage during the cisplatin administration protocol to ensure accurate drug administration. In experiment 1, vehicle or PLX3397 (50 mg/kg) was administered by oral gavage once every 3 days until day 22 of the 42-day cisplatin administration protocol. On this day, we observed repopulation of the cochlear macrophages; therefore, PLX3397 administration frequency was increased to daily (Fig. 1A and fig. S1). Since we had observed some macrophage repopulation in experiment 1, we conducted a second study (experiment 2), in which vehicle or PLX3397 (50 mg/kg) was administered daily throughout the cisplatin administration protocol to ensure that macrophages remained ablated throughout the experiment (Fig. 4). In each experiment, end point auditory tests were conducted at the end of the 42-day cisplatin administration protocol, and PLX3397 administration via oral gavage was continued during the auditory testing phase until mice were euthanized for tissue harvest.

### Auditory testing

Before the start of PLX3397 chow (pretreatment) and at the end of the cisplatin administration protocol, mice underwent two types of auditory tests: ABRs (measure hearing sensitivity) and DPOAEs (indirectly measure OHC function). Mice were anesthetized with ketamine (Putney Inc., Portland, ME; 100 mg/kg, ip) and xylazine (Akorn Inc., Lake Forest, IL; 10 mg/kg, ip). Additional injections at ^1^/_3_ to ^1^/_2_ of the original dose were administered if needed. Animals were placed on a temperature-controlled (37°C) heating pad (World Precision Instruments, T-2000, Sarasota, FL, USA) during auditory testing inside a noise-canceling chamber (Acoustic Systems, Austin, TX, USA). ABRs and DPOAEs were recorded from the left ear of each mouse using Tucker Davis Technologies hardware (RZ6 Processor) and software (BioSigRZ).

For ABR measurements, subcutaneous needle electrodes (Rhythmlink, Columbia, SC, USA) were placed behind left pinna of the test ear (reference), vertex (active), and near the tail of the mouse (ground). Tone-burst stimuli (Cos2, 3 ms, 0.5-ms rise/fall in alternating polarity) were presented at a rate of 29.9/s at 8, 11.2, 16, 22.4, 32, and 40 kHz starting at 90-dB sound pressure level (SPL). At each sound level, 1024 waveforms were averaged, amplified (20×), and filtered [Highpass filter (HP), 300 Hz; Lowpass filter (LP), 3 kHz; Notch filter (NT), 60 Hz]. At near-threshold SPLs, the ABR waveforms were recorded twice, and two waveforms were superimposed for comparison. ABR threshold was defined as the lowest stimulus intensity that resulted in a reproducible waveform displaying identifiable peaks.

DPOAEs were measured in response to two primary pure tones, *f*_1_ and *f*_2_, generated by Multi Field 1 speakers. Two primary tones were presented at six frequency pairs, where *f*_2_ corresponded to ABR test frequencies (*f*_2_ = 8, 11.2, 16, 22.4, 32, and 40 kHz; *f*_2_/*f*_1_ = 1.25). The sound level was increased in 5-dB steps from 30 to 90 dB. At each sound level, 512 responses were averaged. DPOAE at 2*f*_1_ − *f*_2_ was recorded in the mouse inner ear canal using an ER-10B+ microphone (Etymotic, Elk Grove Village, IL, USA) connected to a modified pipette tip to fit the mouse external ear canal. Biological noise floors and amplitudes were calculated for each treatment group and plotted relative to each other.

### Blood and tissue collection

All experimental animals were anesthetized by injecting a cocktail of ketamine (120 mg/kg) and xylazine (25 mg/kg). After achieving surgical level of anesthesia (verified by the lack of hind paw pinch reflex and eyelid reflex), an incision was made along the abdomen. The abdominal cavity was exposed, and the widest part of the inferior vena cava between the kidneys was located. A heparin-coated 28-gauge needle (0.2% heparin solution, 07980, STEMCELL Technology Inc.) in a 1-ml syringe was carefully inserted into the vein to withdraw blood. Blood was transferred into an Eppendorf tube on ice, spun at 3000 rpm for 25 min at 4°C using a refrigerated benchtop centrifuge (Eppendorf centrifuge 5427R), and stored at -80°C until further use.

Following blood collection, cardiac perfusion with 1× phosphate-buffered saline (PBS) (diluted in Milli-Q water from 10× PBS; 70011-044, Thermo Fisher Scientific) was performed. The thoracic cavity was opened by incising through the rib cage and diaphragm, and the heart was exposed. The right atrium was opened by incision; a 23- to 25-gauge butterfly needle was inserted into the left ventricle, and the body was perfused with 1× PBS via the blood flow. After cardiac perfusion, mice were decapitated using surgical scissors, and the inner ears, kidneys, and spleens were collected for further analyses. Inner ears were dissected from the temporal bone; a small hole was opened in the apex of the cochlea using a 27-gauge insulin needle, and the cochlea was perfused with 4% paraformaldehyde (PFA) (diluted from 16% PFA; 5710-S, Electron Microscopy Sciences) through the round window, oval window, and the apical hole for complete fixation of the tissue. Inner ears were then postfixed for 5 to 6 hours at room temperature. Following fixation, inner ears were washed with 1× Dulbecco’s PBS (DPBS) (14190-250, Thermo Fisher Scientific) and decalcified in 0.5 M EDTA solution (pH 8.0) for 26 hours at room temperature. Inner ears were washed again with 1× DPBS and stored at 4°C until dissection. Left kidneys were cut in the horizontal plane into three pieces, frozen immediately in dry ice, and stored at −80°C. Right kidneys were dissected out, and renal capsules were removed and fixed in 10% neutral-buffered formalin and were prepared for histology. Spleens were collected to examine the impact of PLX3397 on peripheral immune cells using flow cytometry.

### Microdissection, cryosectioning, and staining (inner ear)

For wholemount dissections of the cochlea, the stria vascularis was peeled from the cochlear lateral wall. Organ of Corti and SGNs were cut from the surrounding tissues and microdissected into five pieces (apex, mid-apex, mid-base, base, and base-hook) spanning all cochlear regions from base to apex.

For mid-modiolar sections of the inner ear, fixed and decalcified inner ears were cryoprotected in 15% sucrose/DPBS overnight, 20% sucrose/DPBS overnight, and 30% sucrose/DPBS for 2 days at 4°C. Subsequently, inner ears were immersed in 1:1 mixture of 30% sucrose and super cryoembedding medium (C-EM001, Section-Lab Co. Ltd., Hiroshima, Japan) for ~1 hour at room temperature. The tissues were then embedded in 100% super cryoembedding medium within a cryomold biopsy square (NC9806558, Thermo Fisher Scientific) and snap-frozen in 2-methylbutane/dry ice. A cryostat microtome (CM3050S, Leica, Vienna, Austria) was used to cut 14-μm-thick mid-modiolar frozen tissue sections. Inner ear sections were dried overnight at room temperature and stored in −80°C until further processing.

For immunostaining, dissected cochlear or stria vascularis wholemounts were incubated in blocking/permeabilization buffer (0.3% Triton X-100, 3% normal goat serum, and 2% bovine serum albumin in 1× DPBS) for 1 to 2 hours at room temperature. Frozen inner ear sections were rehydrated in PBS and then incubated in blocking buffer. Tissues were probed with primary antibodies (diluted in blocking buffer) at room temperature for 1 hour or overnight at 4°C. Subsequently, the tissues were incubated in species/isotype-matched secondary antibodies conjugated with Alexa Fluor (Thermo Fisher Scientific) or tetramethyl rhodamine isothiocyanate (TRITC) (Southern Biotech) at room temperature and stained for nuclei using Hoechst 33342 (H3570, Thermo Fisher Scientific). Information about the antibodies (target, species, and source) and reagents is in table S1. For CtBP2 and GluR2 staining, cochlear wholemount tissues were incubated in blocking buffer (1% Triton X-100, 5% normal goat serum, and 1% bovine serum albumin in 1× DPBS) at room temperature for 1 hour, followed by primary antibody incubations in antibody dilution buffer (1% Triton X-100 and 1% *N*-hydroxysuccinimide, in 1× DPBS) at 37°C overnight in a humidifying container. Following primary antibody staining, cochlear tissues were incubated in secondary antibodies diluted in blocking buffer and incubated at 37°C for 1 hour (*78*). After immunostaining, tissues were mounted with either VECTASHIELD (H-1000, Vector Biolabs) or ProLong Gold (P36934, Thermo Fisher Scientific) antifade mounting medium onto glass slides, coverslipped, and sealed with nail polish. The stria vascularis was mounted with the marginal cells oriented toward the cover glass.

### Microscopy and data analyses (inner ear)

For cochlear wholemounts, low-magnification confocal images of microdissected cochlear pieces were taken and imported to ImageJ (version 2.1.0, Fiji, NIH, Bethesda, MD). Cochlear length was measured to subsequently convert cochlear locations to cochlear frequencies using ImageJ/Plugin/Tools/Measure_line.class (downloaded from www.masseyeandear.org/research/otolaryngology/eaton-peabody-laboratories/histology-core) (*79*). The cochlear frequency map was used to acquire high-resolution confocal z-stack images (Airyscan) from specific cochlear regions corresponding to frequencies of 8, 11.2, 16, 22.4, 32, 40, and 63 kHz along the basilar membrane in each cochlea. High-resolution confocal z-stack images (step size of 0.6 μm) of hair cells and synapses were acquired using a 63× [1.4–numerical aperture (NA) Oil DIC M27] Plan-Apochromat oil-immersion objective lens with a 0.9× optical zoom at a resolution of 2048 pixels by 2048 pixels, with a scanning speed of 2.05 μs per pixel. The imaging was performed on an Axiovert 200M inverted microscope with a confocal scan head (LSM980, Carl Zeiss Microscopy) equipped with an Airyscan detection unit, all controlled by Zen Blue v3.4 software.

For SGN and macrophage quantification in cochlear sections, images encompassing the entire cross section of the cochlea were obtained with a Zeiss LSM980 microscope through a series of overlapping images (six or eight tiles, 10% overlap). Subsequently, these tiled images were stitched together to generate a single image of the whole cochlear section, through the tiling and stitching function in the Zen Blue v3.4 software. Images were acquired using a Plan-Apochromat 20× (0.8-NA M27) objective lens at a resolution of 1024 pixels by 1024 pixels, with a scanning speed of 0.51 μs per pixel. The stria vascularis wholemounts were imaged using a 20× objective lens (0.8 NA) with a 1.1× optical zoom at a resolution of 2048 pixels by 2048 pixels, with a scanning speed of 2.05 μs per pixel (Airyscan super-resolution).

OHCs, IHCs, SGNs, and macrophages were manually counted using the ImageJ software (ImageJ/Point tool) (NIH, Bethesda, MD, USA). IHC synapses were semiautomatically quantified using Imaris ×64 9.2.1 and Imaris File Converter ×64 9.1.2 (Oxford Instruments, Abingdon, Oxfordshire, England). OHCs, IHCs, and IHC synapses were quantified within the span of 147.90 μm. The total numbers of synaptic puncta (CtBP2 and GluR2 staining) were divided by the number of IHCs to gain the number of puncta associated with each IHC. SGNs in Rosenthal’s canal were quantified from mid-modiolar sections of the inner ear, and the total numbers of SGNs were normalized to the area of Rosenthal’s canal to obtain density measurements (SGNs per 10,000 μm^2^). The SGN density from each of the three regions (apex, mid, and base) of the cochlea were averaged separately. Macrophages in the osseous spiral lamina of cochlear wholemounts were quantified within a 180-μm by 180-μm region of interest. PVMs in the stria vascularis wholemounts were quantified in the entire image frame (20× objective zoom and 1.1× digital zoom; Airyscan; 2024 pixels by 2024 pixels).

### Morphological evaluation (kidney)

After harvesting under anesthesia, kidney specimens were fixed with 10% formalin and subsequently embedded in paraffin. To assess tubular injury, sections (4 μm in thickness) were stained with periodic acid–Schiff, while Masson’s trichrome reagent was used to evaluate renal fibrosis. Tubular injury and interstitial fibrosis were evaluated by a blinded observer in 10 randomly selected nonoverlapping fields at ×400 magnification in each section of corticomedullary junction. This region contains proximal tubule S3 segments, which are the most vulnerable to cisplatin-induced injury (*80*). As previously described (*67*), tubular injury judged by tubular atrophy, tubular dilation, protein casts, tubular necrosis, and brush border loss was rated on the following scoring system: 0, 0%; 1, 1 to 25%; 2, 26 to 50%; 3, 51 to 75%; 4, 76 to 100%. The fibrotic area was calculated using Fiji/ImageJ software (NIH, Bethesda, MD, USA).

### Immunohistochemistry (kidney)

Formalin-fixed paraffin-embedded kidney tissues were sectioned, deparaffinized, and subjected to antigen retrieval in sodium citrate buffer [10 mM sodium citrate and 0.05% Tween 20 (pH 6.0)] for 20 min at 98°C using a microwave. Endogenous peroxidase activity was blocked with 0.3% hydrogen peroxide in methyl alcohol for 15 min. After blocking with goat serum, the specimens were incubated overnight at 4°C with rabbit anti-GFP antibody (5 μg/ml; ab290, Abcam, Cambridge, MA, USA). Subsequently, horseradish peroxidase–conjugated goat anti-rabbit antibody (Agilent Dako, Santa Clara, CA, USA) was applied to sections and incubated for 1 hour at room temperature. Sections were developed using 3,3′-diaminobenzidine tetrahydrochloride (Sigma-Aldrich, St. Louis, MO, USA) and then counterstained with hematoxylin. Positively stained cells were counted in 10 randomly selected nonoverlapping fields at ×400 magnification of corticomedullary junction.

### Measurement of kidney function and injury marker

BUN was measured from 2 μl of plasma using a Quantichrom Urea Assay Kit (Bioassay System, Hayward, CA, USA). Plasma NGAL was assessed with enzyme-linked immunosorbent assay (R&D Systems, Minneapolis, MN, USA) according to the manufacturer’s instructions.

### Quantification of mRNA by reverse transcription PCR (kidney)

For quantification of RNA transcripts, total RNA was isolated from kidney tissues (~20 mg) using E.Z.N.A Total RNA kit (R6812-02, Omega Bio-tek) with modifications of the manufacturer’s protocol. Invitrogen SuperScript III First strand synthesis (18080051, Thermo Fisher Scientific) was used to synthesize cDNA from total RNA. TaqMan Fast Advanced Master Mix (4444557, Thermo Fisher Scientific) was used to detect *Tnf* (Mm00443258_m1), *Tgfb1* (Mm01178820_m1), *Tlr2* (Mm00442346_m1), and *Tlr4* (Mm00445273_m1) (Invitrogen). The fluorescein amidites (FAM) and minor groove binder (MGB) were used as the reporter dye and the quencher, respectively. Multiplex reverse transcription PCR assay was performed to simultaneously detect gene of interest and *Gapdh* (Mm9999991591) as an internal control gene, using Victoria and MGB_PL as the reporter dye and the quencher. Fold change was expressed as 2^–ΔΔ*C*t^. Values of experimental groups were normalized to values derived from the saline/vehicle-treated group.

### Inductively coupled plasma mass spectrometry

ICP-MS was used to measure platinum content in biological samples. Cisplatin contains a platinum atom at its core, and, therefore, measurement of platinum is an indication of cisplatin content. The cochlear tissues were microdissected into stria vascularis, spiral ligament, organ of Corti, and SGNs; vestibular organs were microdissected into utricle, saccule, and cristae. Dissected tissues were submerged in UltraPure Distilled Water (10977023, Invitrogen, Waltham, MA) during dissection. Liquid was removed via a speed vacuum concentrator (Eppendorf Vacufuge) and kept frozen at −80°C for experiment 1 samples and kept at room temperature for experiment 2 samples until ready for analysis.

As previously mentioned, during mouse tissue harvest, left kidneys were cut in the horizontal plane into three pieces, frozen immediately in dry ice, and stored at −80°C. The middle piece of the fresh frozen kidneys, which included the renal artery and vein, was immersed in 4% PFA (diluted with 1× DPBS from 16% PFA) and fixed overnight at 4°C. Subsequently, kidney samples were washed three times in 1× DPBS, dabbed on a paper towel to absorb excess fluid, and transferred to new centrifuge tubes for sample submission.

ICP-MS was performed at the Mass Spectrometry Core Facility at the University of Massachusetts Amherst using NexION 350D ICP-MS (PerkinElmer, Waltham, MA). Organs were digested in 50 μl (cochlear tissues) or 100 μl (kidney tissues) of trace metal grade nitric acid (HNO_3_) and incubated for 20 min at 65°C. An equal volume of hydrogen peroxide (Optima Grade, Fisher Scientific) was added, and the incubation was repeated for 20 min at 65°C. Samples were spun down for 1 min at 14,000*g* and diluted 1:20 with Milli-Q water before ICP-MS measurements. Platinum (Pt; molecular weight, 195) and sulfur monoxide (SO; molecular weight, 48) were measured in dynamic reaction mode using oxygen at a gas flow rate of 1.2 ml/min. Platinum [0.5 parts per thousand to 10 parts per billion (ppb) standards] and sulfur (50 ppb to 500 ppb standards) standard curves were generated using single element standards (PerkinElmer) to quantitatively measure levels of platinum and sulfur. Platinum levels were normalized to sulfur levels for each sample.

### Flow cytometry

Spleens were collected, cut in smaller pieces, and placed in media containing RPMI (112-025-101, Quality Biologicals), 5% heat-inactivated fetal bovine serum (10082-147, Thermo Fisher Scientific), and 0.5% penicillin (P3032, Sigma-Aldrich). Tissues were mechanically disrupted with a 500-μl syringe plunger and passed through a 40-μm cell strainers (BD Falcon, San Jose, CA, USA) to obtain single-cell suspension. RBC lysis was performed at room temperature for 5 min in 1× RBC lysis buffer (10× RBC lysis buffer diluted in Milli-Q water; 420301, BioLegend), and reaction was stopped by diluting the 1× RBC lysis buffer with 35 ml of 1× DPBS. Cells were resuspended in fluorescence-activated cell sorting buffer (1× DPBS, 5% heat-inactivated fetal bovine serum, and 2 mM EDTA) and counted (Cellometer K2, Nexcelom, Lawrence MA). Spleen single-cell suspensions were pretreated with anti-mouse CD16/CD32 antibody (101319, BioLegend) for blocking of Fc receptors, followed by surface staining with antibodies in fluorescence-activated cell sorting buffer for 30 min at 4°C. Information about the antibodies is presented in table S1. Cells were then washed and incubated in Fixable Viability dye eFluor450 (65-0863, eBioscience) at a 1:1000 dilution in 1× DPBS and incubated for 30 min at 4°C. Cells were washed again, and flow cytometric acquisition was performed on a BD LSRFortessa (BD Biosciences, Franklin Lakes, NJ). Data were analyzed using FlowJo software version 10 (BD Biosciences, Ashland, OR). Cells were initially gated on forward scatter, side scatter, and viability dye to eliminate cell debris/dead cells and gate on live cell populations. Live cells were gated for CD45 expression, a pan-leukocyte marker. Cell surface markers were used to identify specific populations of immune cells, including macrophages (CD11b^+^, CD11b^+^ CX3CR1^GFP+^, and CD11b^+^ CX3CR1^GFP−^), neutrophils (CD11b^+^ F4/80^−^ Ly6G^+^), NK cells (CD3e^−^ NK1.1^+^), T cells (CD11b^−^ CD3e^+^), and B cells (CD11b^−^ CD19^+^).

### Statistics

Statistical analyses were carried out using GraphPad Prism 9 (GraphPad, San Diego, CA). The Shiapiro-Wilks normality test was used to verify the normal distribution of the data. A Student’s *t* test was performed to compare two groups. Comparisons of multiple groups were conducted using either ordinary one-way analysis of variance (ANOVA), followed by Tukey’s post hoc multiple comparisons test or two-way ANOVA to detect the main effect of PLX3397 treatment across multiple groups.

For statistical analysis to assess the protective effect of PLX3397 on male and female mice in experiment 1, mixed-effects modeling was performed in R (version 4.1.3) with the “lmerTest” package. Confidence intervals and *P* values were calculated using the *t*-distribution with degrees of freedom determined via Satterthwaite’s method. Incorporating “animal” as a random effect in the model assumes that each mouse responds differently to the treatment. To evaluate differences in the protection provided by PLX3397 treatment between males and females, an interaction term “Treatment * Sex” was introduced, and the following model was used

$$\begin{array}{c} \text{DPOAE}=\text{Level}+\text{Treatment}+\text{Sex}+{\text{Treatment}}^{*}\;\text{Sex}\\ +\left(1|\text{Animal}\right) \end{array}$$

Data are presented as means ± SD or means ± SEM. Statistical significance is indicated as the following: *P < 0.05, **P < 0.01, ***P < 0.001, and ****P < 0.0001. *P* value above 0.05 (*P* > 0.05) was considered nonsignificant and is denoted as “n.s.” Statistical comparisons are presented in color-coded remarks (n.s. or asterisks) for Figs. 1B; 2, A to C; and 4B and fig. S13B. Saline/vehicle group is compared to saline/PLX3397 (purple remarks), cisplatin/vehicle (red remarks), and cisplatin/PLX3397 (green remarks). Comparisons between the cisplatin/vehicle and cisplatin/PLX3397 groups are denoted in black remarks.
