# Supplementary material for: Macrophage depletion protects against cisplatin-induced ototoxicity and nephrotoxicity
Source: Sci Adv. 2024 Jul 24;10(30):eadk9878. doi: 10.1126/sciadv.adk9878 (PMC11268410; doi:10.1126/sciadv.adk9878)
Supplement: Supplementary file 1 — Figs. S1 to S16 Table S1 [file sciadv.adk9878_sm.pdf]

Science Advances
AAAS

Supplementary Materials for

Macrophage depletion protects against cisplatin-induced ototoxicity and nephrotoxicity

Cathy Yea Won Sung *et al.*

Corresponding author: Cathy Yea Won Sung, cathy.sung@nih.gov; Lisa L. Cunningham, lisa.cunningham@nih.gov

Sci. Adv. **10**, eadk9878 (2024)
DOI: 10.1126/sciadv.adk9878

This PDF file includes:

Figs. S1 to S16
Table S1

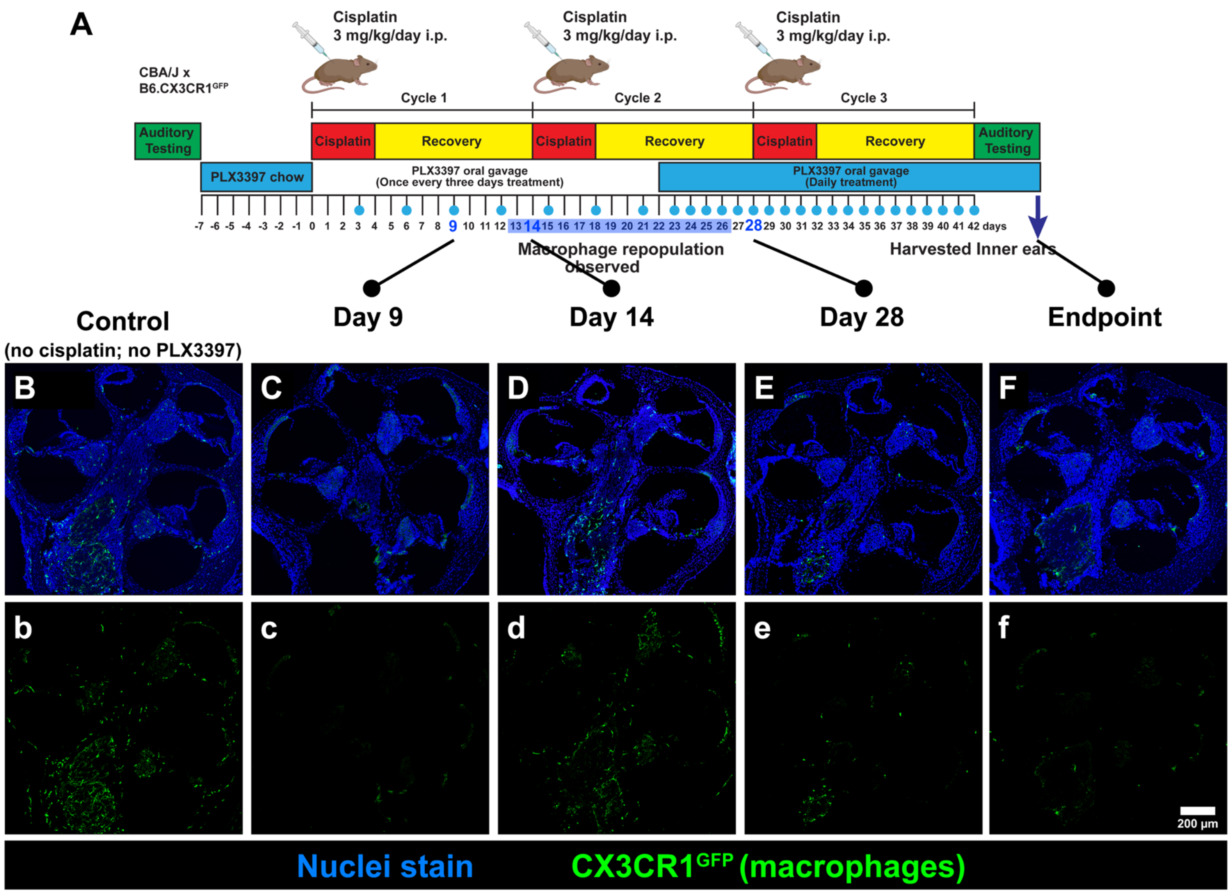

A
CBA/J x B6.CX3CR1GFP
Cisplatin 3 mg/kg/day i.p.
Cycle 1
Cycle 2
Cycle 3
Auditory Testing
Cisplatin
Recovery
PLX3397 chow
PLX3397 oral gavage (Once every three days treatment)
PLX3397 oral gavage (Daily treatment)
days
Macrophage repopulation observed
Harvested Inner ears
Day 9
Day 14
Day 28
Endpoint
Control
(no cisplatin; no PLX3397)
B
C
D
E
F
b
c
d
e
f
200 µm
Nuclei stain
CX3CR1GFP (macrophages)

Fig. S1. PLX3397 treatment once every three days resulted in partial macrophage repopulation (Experiment 1). (A) Following baseline auditory testing, mice received either control chow or PLX3397-formulated chow for seven days. Subsequently, PLX3397 was administered via oral gavage once every three days, followed by daily dosing initiated after the midpoint of cycle 2 and continued until mice were euthanized after the endpoint auditory testing. (B-F, b-f) Cochleae from intermediate time points were harvested to visualize CX3CR1^{GFP}-positive macrophages. Nuclei were stained with Hoechst 33342 (blue). (B,b) Control cochleae were not exposed to cisplatin or PLX3397. (C,c) Macrophage ablation was efficient at Day 9. (D,d) Macrophage repopulation was observed at Day 14. (E,e; F,f) To address this, the frequency of PLX3397 administration was increased to daily, resulting in subsequent macrophage ablation by day 28. The days on which mice received PLX3397 oral gavage treatment are marked by blue circles in the experimental timeline. Scale bar, 200 μm.

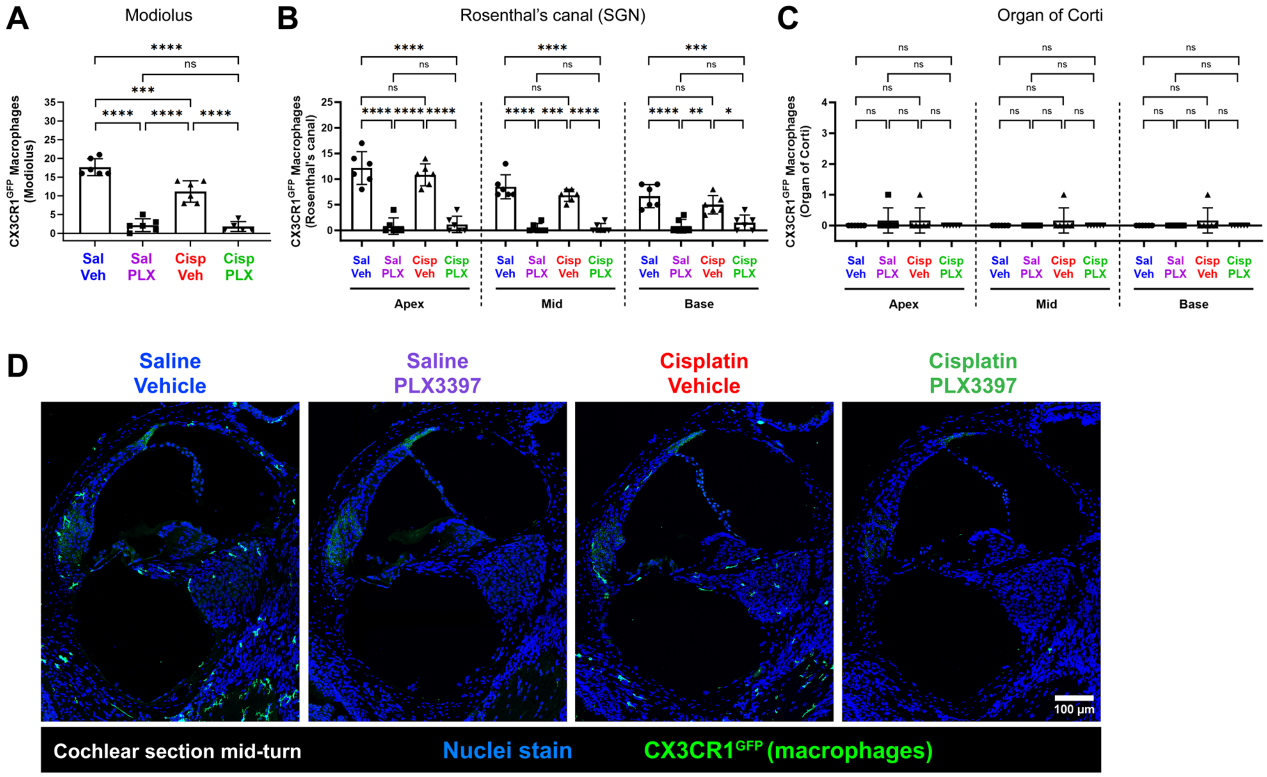
A
Modiolus
CX3CR1GFP Macrophages (Modiolus)
Sal Veh
Sal PLX
Cisp Veh
Cisp PLX
B
Rosenthal's canal (SGN)
CX3CR1GFP Macrophages (Rosenthal's canal)
Apex
Mid
Base
C
Organ of Corti
CX3CR1GFP Macrophages (Organ of Corti)
D
Saline Vehicle
Saline PLX3397
Cisplatin Vehicle
Cisplatin PLX3397
100 µm
Cochlear section mid-turn
Nuclei stain
CX3CR1GFP (macrophages)

Fig. S2. Macrophage ablation followed by partial repopulation resulted in comparable depletion of macrophages in various compartments within the cochlea (Experiment 1). After completing three cycles of the cisplatin administration protocol with initial PLX3397 treatment through once-in three days oral gavage, followed by subsequent endpoint auditory testing, cochleae were harvested. $CX3CR1^{GFP}$-positive macrophages were quantified in various compartments within the cochlea, including (A) modiolus, (B) Rosenthal's canal, and (C) organ of Corti. (A-C) Statistical analysis was performed using one-way ANOVA with Tukey's multiple comparisons test. (D) Representative images of the middle turn of the cochlea showing macrophage density and distribution in different cochlear compartments in the four treatment groups: Saline/Vehicle, Saline/PLX3397, Cisplatin/Vehicle, and Cisplatin/PLX3397. Nuclei were stained with Hoechst 33342 (blue). Scale bar, 100 µm.

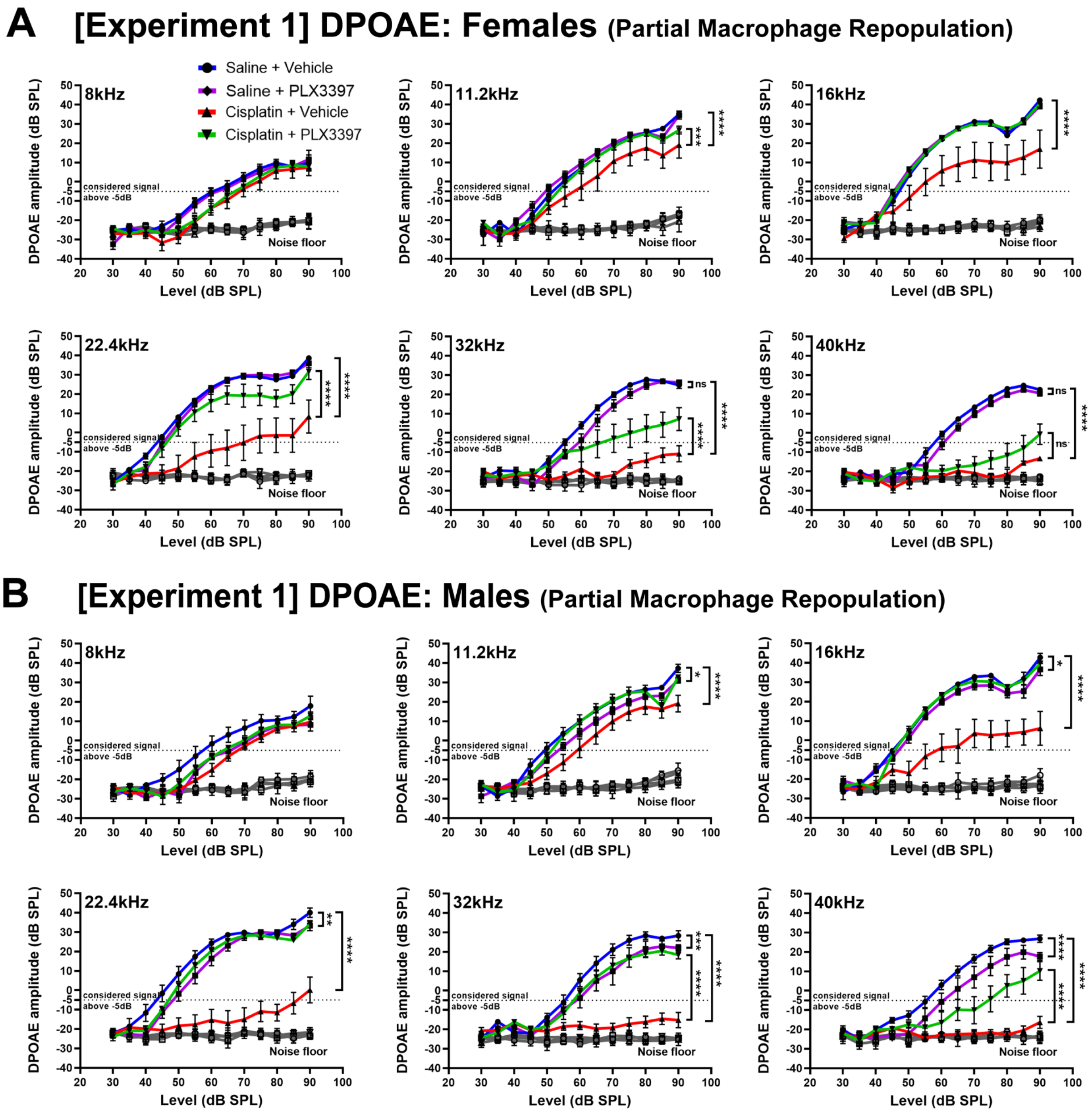
A
[Experiment 1] DPOAE: Females (Partial Macrophage Repopulation)
Saline + Vehicle
Saline + PLX3397
Cisplatin + Vehicle
Cisplatin + PLX3397
8kHz
11.2kHz
16kHz
22.4kHz
32kHz
40kHz
DPOAE amplitude (dB SPL)
Level (dB SPL)
considered signal
above -5dB
Noise floor
ns
B
[Experiment 1] DPOAE: Males (Partial Macrophage Repopulation)

Fig. S3. Macrophage ablation followed by partial repopulation resulted in protection against OHC dysfunction in mice of both sexes (Experiment 1). DPOAEs were recorded to assess OHC function in female and male mice. An emission at $2f_1$-f_2 was considered present when its amplitude exceeded -5dB (dotted line). Sample groups are represented by color-coded line: saline/vehicle (blue line), saline/PLX3397 (purple line), cisplatin/vehicle (red line), and cisplatin/PLX3397 (green line). (A) female and (B) male mice. Grey line indicates the biological noise floor. Statistical analyses were performed using two way-ANOVA with Tukey's multiple comparisons test (main column effect). Mean±SEM, n=10-16 mice (5-7F and 5-9M) per experimental group (total 49 mice; 24F and 25M). Statistical analyses to identify the difference in protection between males and females were performed using Mixed Effect Modeling with the R package "lmerTest" as described in the Methods.

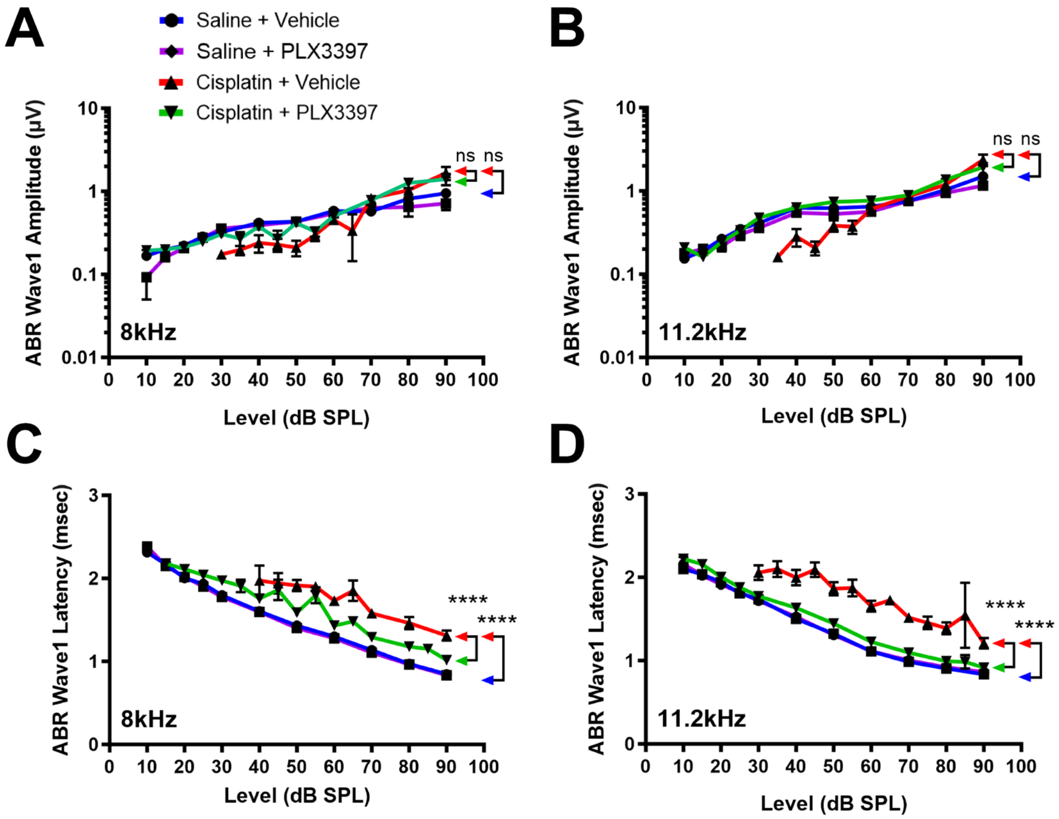

A
B
C
D
Saline + Vehicle
Saline + PLX3397
Cisplatin + Vehicle
Cisplatin + PLX3397
ABR Wave1 Amplitude (µV)
ABR Wave1 Latency (msec)
Level (dB SPL)
8kHz
11.2kHz
ns
****

Fig. S4. Co-administration of cisplatin and PLX3397 protects against cisplatin-induced impairment of wave I latency (Experiment 1). (A-D) Auditory sensitivity was measured by ABRs at baseline and endpoint and wave I amplitude and latency were assessed at low frequencies at (A,C) 8kHz and (B,D) 11.2kHz.Statistical analyses were performed using two way-ANOVA with Tukey's multiple comparisons test (main column effect). Mean±SEM, n=10-15 mice (4-6F and 5-9M) per experimental group (total 46 mice; 21F and 25M).

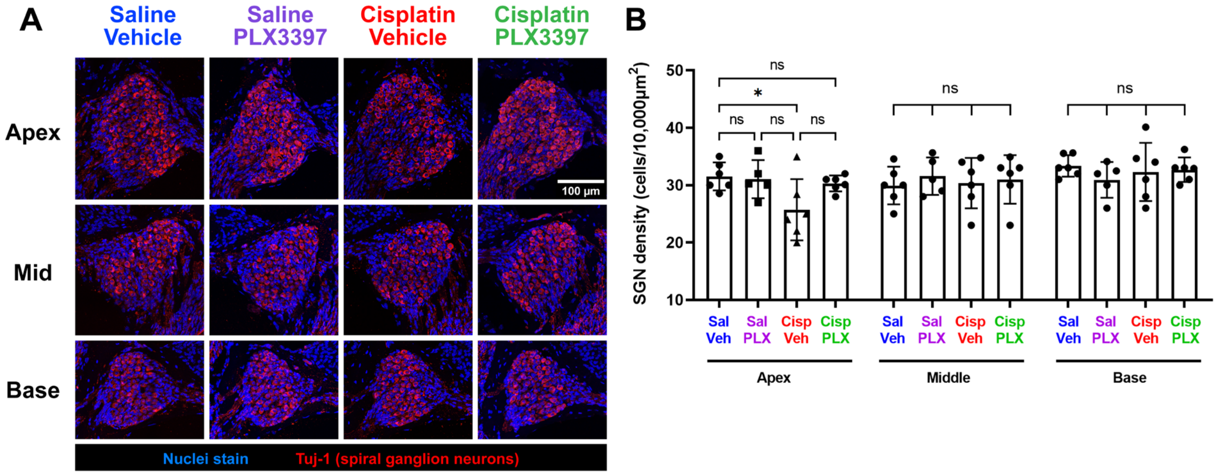

A
Saline Vehicle
Saline PLX3397
Cisplatin Vehicle
Cisplatin PLX3397
Apex
Mid
Base
100 μm
Nuclei stain
Tuj-1 (spiral ganglion neurons)
B
SGN density (cells/10,000μm²)
50
40
30
20
10
ns
*
Sal Veh
Sal PLX
Cisp Veh
Cisp PLX
Apex
Middle
Base

Fig. S5. Macrophage ablation followed by partial repopulation using PLX3397 protects against cisplatin-induced SGN loss (Experiment 1). Inner ear tissues were harvested following endpoint auditory testing and stained for Tuj-1 (red) to visualize and quantify SGNs. Nuclei were stained with Hoechst 33342 (blue). (A) Representative images and (B) quantification demonstrate that cisplatin-induced SGN loss occurred primarily in the apex, with no significant loss in the middle or basal regions. PLX3397 protected against cisplatin-induced loss of SGNs. (A) Scale bar, 100 µm. (B) Mean±SD, n=6 cochleae (3F and 3M) per experimental group (total 24 cochleae; 12F and 12M). Statistical analysis was performed using one way-ANOVA with Tukey's multiple comparisons test.

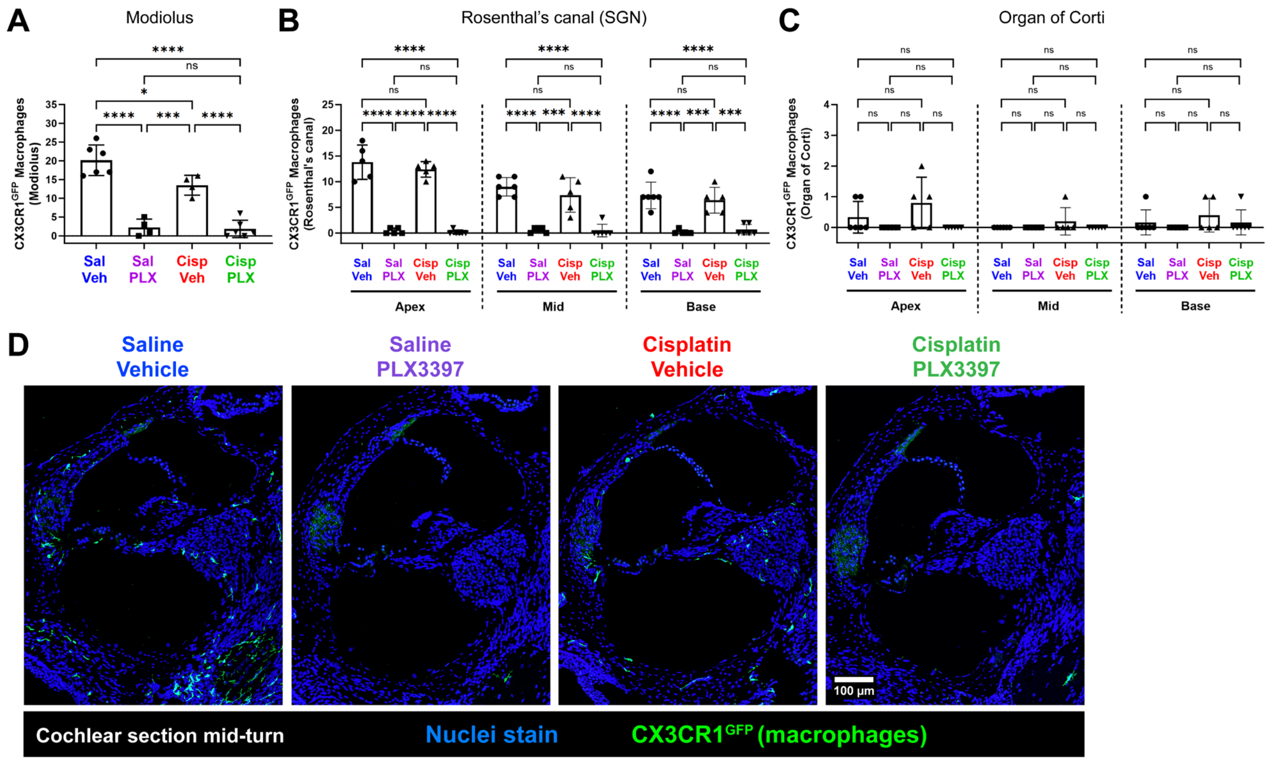
A
Modiolus
CX3CR1GFP Macrophages (Modiolus)
Sal Veh
Sal PLX
Cisp Veh
Cisp PLX
B
Rosenthal's canal (SGN)
CX3CR1GFP Macrophages (Rosenthal's canal)
Apex
Mid
Base
C
Organ of Corti
CX3CR1GFP Macrophages (Organ of Corti)
D
Saline Vehicle
Saline PLX3397
Cisplatin Vehicle
Cisplatin PLX3397
100 µm
Cochlear section mid-turn
Nuclei stain
CX3CR1GFP (macrophages)

Fig. S6. Sustained macrophage ablation resulted in comparable depletion of macrophages in various compartments within the cochlea (Experiment 2). Following three cycles of cisplatin administration protocol with daily oral gavage of PLX3397, and subsequent auditory testing at the endpoint, cochleae were collected. CX3CR1^{GFP}-positive macrophages were visualized and quantified in various compartments within the cochlea including (A) modiolus, (B) Rosenthal's canal, and (C) organ of Corti. (A-C) Statistical analysis was performed using one-way ANOVA with Tukey's multiple comparisons test. (D) Representative images of the middle turn of the cochlea showing macrophage density and distribution in different cochlear compartments in the four treatment groups: Saline/Vehicle, Saline/PLX3397, Cisplatin/Vehicle, and Cisplatin/PLX3397. Nuclei were stained with Hoechst 33342 (blue). Scale bar, 100 μm.

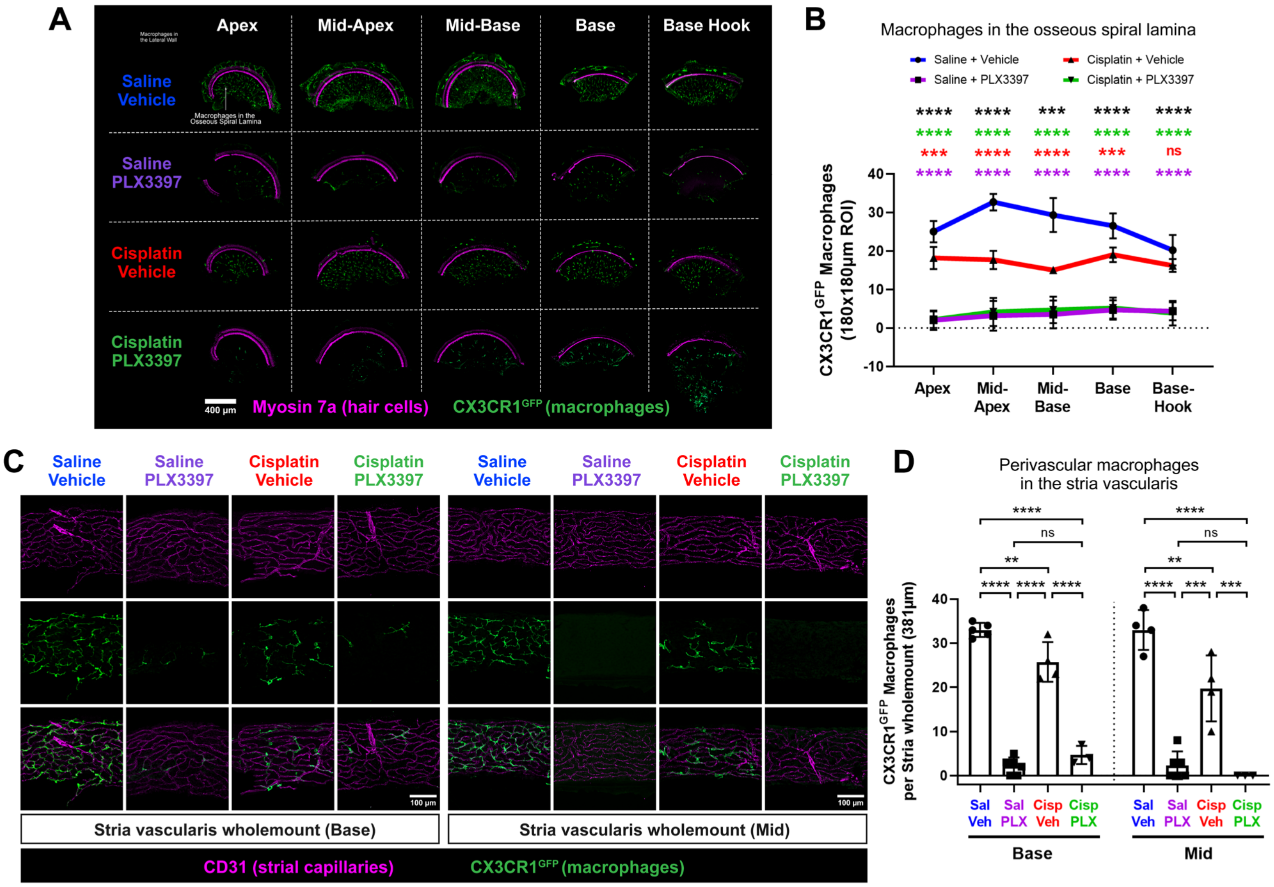

A
Apex
Mid-Apex
Mid-Base
Base
Base Hook
Saline Vehicle
Saline PLX3397
Cisplatin Vehicle
Cisplatin PLX3397
400 μm
Myosin 7a (hair cells)
CX3CR1GFP (macrophages)
B
Macrophages in the osseous spiral lamina
Saline + Vehicle
Cisplatin + Vehicle
Saline + PLX3397
Cisplatin + PLX3397
CX3CR1GFP Macrophages (180x180μm ROI)
Apex
Mid-Apex
Mid-Base
Base
Base-Hook
C
Saline Vehicle
Saline PLX3397
Cisplatin Vehicle
Cisplatin PLX3397
100 μm
Stria vascularis wholemount (Base)
Stria vascularis wholemount (Mid)
CD31 (strial capillaries)
CX3CR1GFP (macrophages)
D
Perivascular macrophages in the stria vascularis
CX3CR1GFP Macrophages per Stria wholemount (381μm)
Sal Veh
Sal PLX
Cisp Veh
Cisp PLX
Base
Mid

Fig. S7. Sustained macrophage ablation using PLX3397 ablates macrophages in the osseous spiral lamina and the stria vascularis (Experiment 2). (A) Representative images and (B) quantitative analysis indicate that daily administration of PLX3397 in Experiment 2 resulted in ablation of macrophages in the osseous spiral lamina overall with the greatest ablation efficiency (92%) in the apex and 80.5% ablation in the basal region of the cochlea, when compared to mice treated with saline/vehicle. In addition, quantitative analysis indicated a significant reduction in macrophages following cisplatin treatment (cisplatin/vehicle-treated mice) compared to mice treated with saline/vehicle, with the middle regions exhibiting the greatest loss (Apex: 27.3%; Mid-Apex: 45.9%; Mid-Base: 48.9%; Base: 28.3%; Base-Hook: 18.9% reduction). Scale bar, 400 μm. Mean±SD, n=6 cochleae (3F and 3M) per experimental group (total 24 cochleae; 12F and 12M). Statistical analysis was performed using one-way ANOVA with Tukey's multiple comparisons test. Statistical comparisons (asterisks or n.s.) are color-coded as described in Methods. (C-D) Stria vascularis wholemounts were dissected from basal and middle regions of the cochlear lateral wall. (C) Representative images and (D) quantitative analysis of $CX3CR1^{GFP}$-positive PVMs in the stria vascularis. Scale bar, 100 μm. (B,D) Mean±SD, n=3-6 cochleae (2-3F and 2-3M) per experimental group (total 19 cochleae; 9F and 10M). Statistical analysis was performed using one-way ANOVA with Tukey's multiple comparisons test.

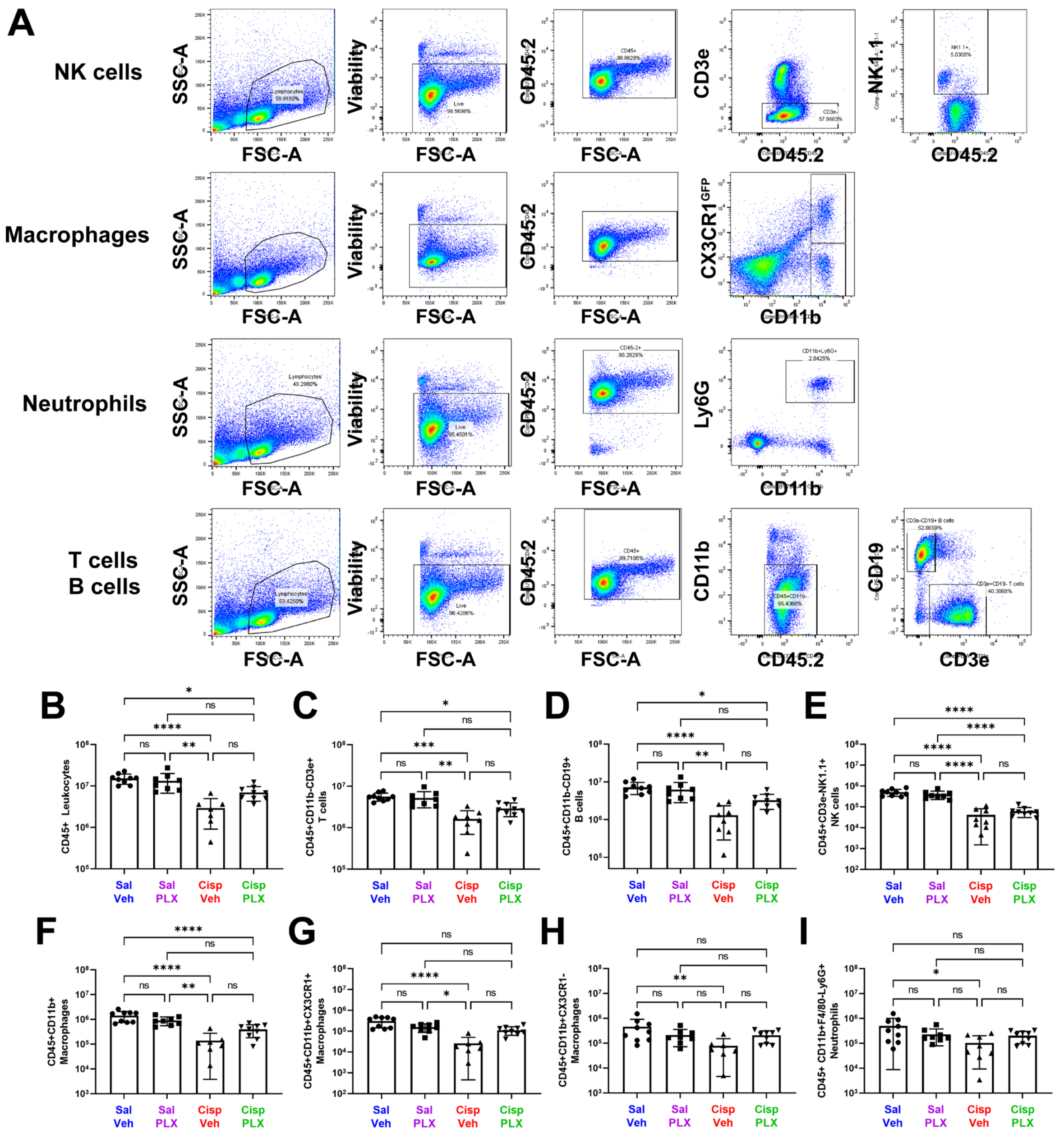
A
NK cells
Macrophages
Neutrophils
T cells
B cells
SSC-A
FSC-A
Viability
CD45.2
CD3e
NK1.1
CX3CR1GFP
CD11b
Ly6G
CD19
B
C
D
E
F
G
H
I
CD45+ Leukocytes
CD45+CD11b-CD3e+ T cells
CD45+CD11b-CD19+ B cells
CD45+CD3e-NK1.1+ NK cells
CD45+CD11b+ Macrophages
CD45+CD11b+CX3CR1+ Macrophages
CD45+CD11b+CX3CR1- Macrophages
CD45+ CD11b+F4/80-Ly6G+ Neutrophils
Sal Veh
Sal PLX
Cisp Veh
Cisp PLX
ns
*
**
***
****

Fig. S8. CSF1R inhibition via PLX3397 treatment does not affect peripheral immune cells in the spleen (Experiment 2). Spleens were isolated and single cell suspensions were used for flow cytometric analyses. (A) A representative gating strategy was used to analyze immune cell profiling in the spleen. The absolute numbers of immune cells in the spleen were analyzed: (B) CD45+ Leukocytes, (C) T cells, (D) B cells, (E) NK cells, (F) CD11b+ macrophages, (G) CD11b+ CX3CR1+ macrophages, (H) CD11b+ CX3CR1- macrophages, and (I) neutrophils. Cisplatin reduced the numbers of all analyzed leukocytes. Sustained macrophage ablation via PLX3397 administration did not significantly alter the numbers of leukocytes in the spleen. Mean±SD, n=7-9 spleens (3-4F and 4-6M) per experimental group (total 34 spleens; 14F and 20M). Statistical analysis was performed using Kruskal-Wallis test with Dunn’s multiple comparisons test.

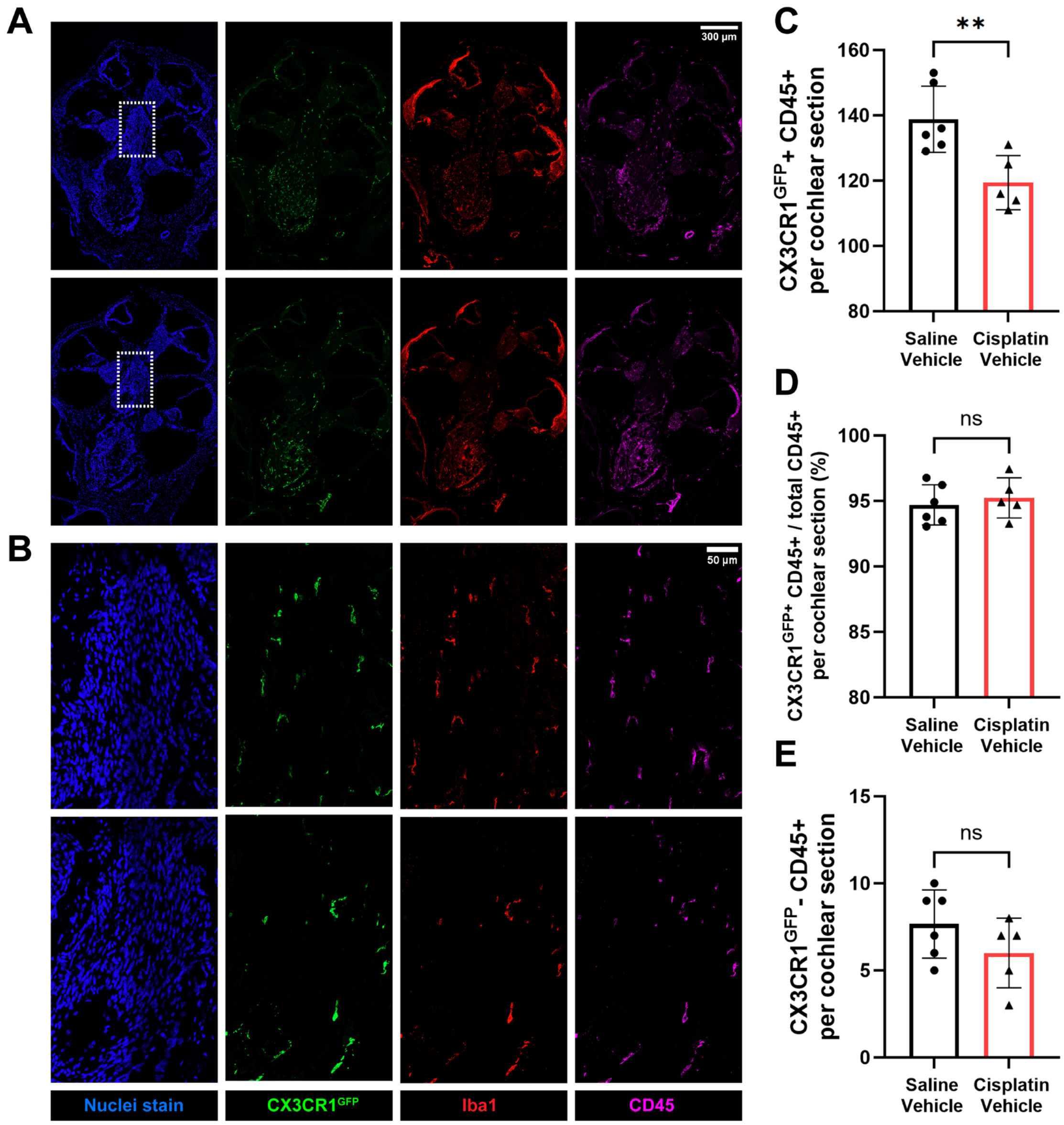
A
B
300 µm
50 µm
Nuclei stain
CX3CR1GFP
Iba1
CD45
C
**
CX3CR1GFP+ CD45+
per cochlear section
160
140
120
100
80
Saline Vehicle
Cisplatin Vehicle
D
ns
CX3CR1GFP+ CD45+ / total CD45+
per cochlear section (%)
100
95
90
85
80
Saline Vehicle
Cisplatin Vehicle
E
ns
CX3CR1GFP- CD45+
per cochlear section
15
10
5
0
Saline Vehicle
Cisplatin Vehicle

Fig. S9. Cisplatin does not induce macrophage activation and immune cell infiltration into the cochlea (Experiment 2). Inner ear tissues were harvested following endpoint auditory testing and stained for CX3CR1^{GFP} (green), Iba-1 (red), and CD45 (magenta). Iba-1 expression, known to increase with macrophage activation, was visualized to assess the activation state of cochlear macrophages. Representative image of (A) the whole cochlear section (*Scale bar, 300 µm)* and (B) magnified inset images (from white box in panel A) of the cochlear modiolus *(Scale bar, 50 µm)*. Nuclei were stained with Hoechst 33342 (blue). (C-E) Quantification of CX3CR1^{GFP} and CD45 labeling was performed to assess (C) the number of [CX3CR1^{GFP}+CD45+] macrophages, (D) the percentage of [CX3CR1^{GFP}+CD45+] macrophages to total number of CD45+ cells ((CX3CR1^{GFP}+CD45+ / total CD45+))x100), and (E) the number of infiltrating immune cells into the cochlea by determining the number of cells that are [CX3CR1^{GFP}- CD45+]. Mean±SD, n=6 cochleae (2F and 3-4M) per experimental group (total 11 cochleae; 4F and 7M). Statistical analysis was performed using a Student's t-test.

A [Experiment 2] DPOAE: Females

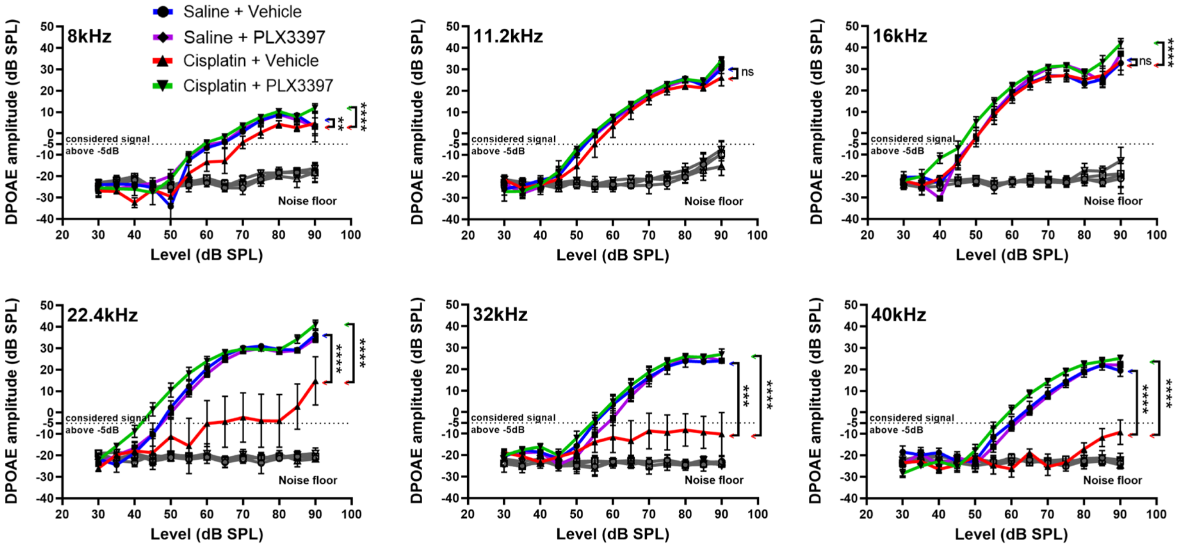

B [Experiment 2] DPOAE: Males

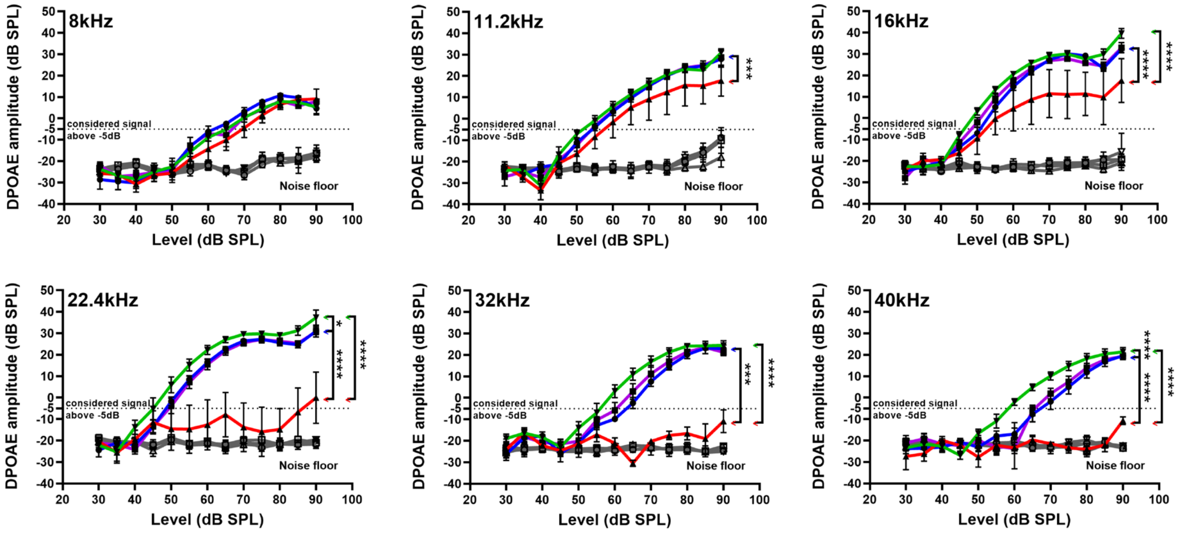

Fig. S10. Sustained macrophage ablation using PLX3397 resulted in complete protection against cisplatin-induced OHC dysfunction in both male and female mice (Experiment 2). OHC function was evaluated by DPOAE in (A) female and (B) male mice. DPOAEs were considered present at $2f_1$-f_2 when the DPOAE amplitude surpassed the -5dB threshold (dotted line). The grey line represents the biological noise floor. Data are shown as Mean±SEM, n=8-9 mice (4-5F and 4-5M) per experimental group (total 34 mice; 16F and 16M). Statistical analysis was performed using two way-ANOVA with Tukey's multiple comparisons test (main column effect).

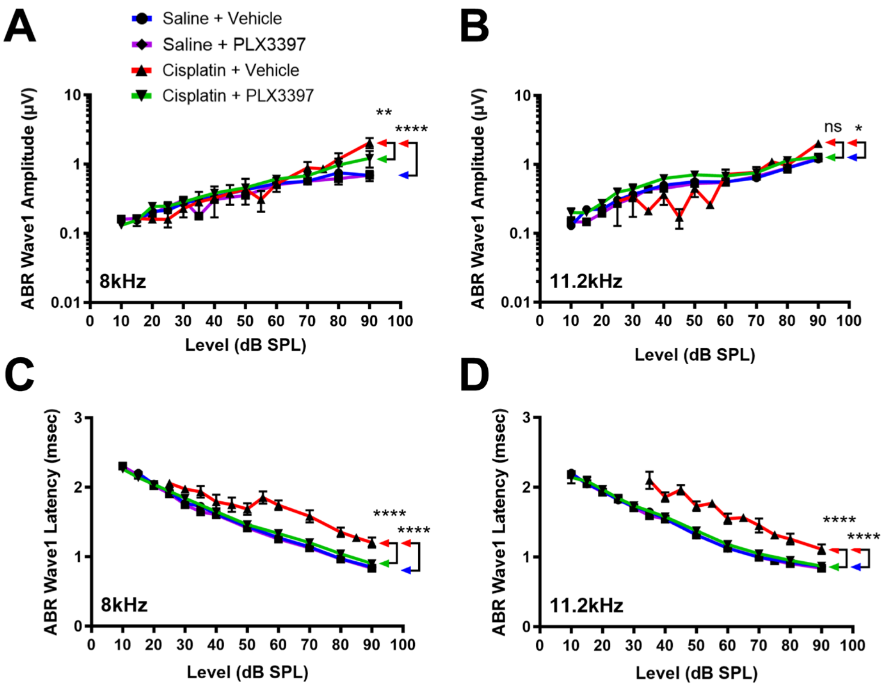
A
B
C
D
Saline + Vehicle
Saline + PLX3397
Cisplatin + Vehicle
Cisplatin + PLX3397
ABR Wave1 Amplitude (μV)
ABR Wave1 Latency (msec)
Level (dB SPL)
8kHz
11.2kHz
**
****
ns
*

Fig. S11. Sustained macrophage ablation protected against cisplatin-induced impairment of wave I latency (Experiment 2). (A-D) Auditory sensitivity was measured by ABRs at baseline and endpoint and wave I amplitude and latency were assessed at low frequencies at (A,C) 8kHz and (B,D) 11.2kHz. Statistical analyses were performed using two way-ANOVA with Tukey's multiple comparisons test (main column effect). Mean±SEM, n=7-9 mice (3-4F and 4-5M) per experimental group (total 32 mice; 14F and 18M).

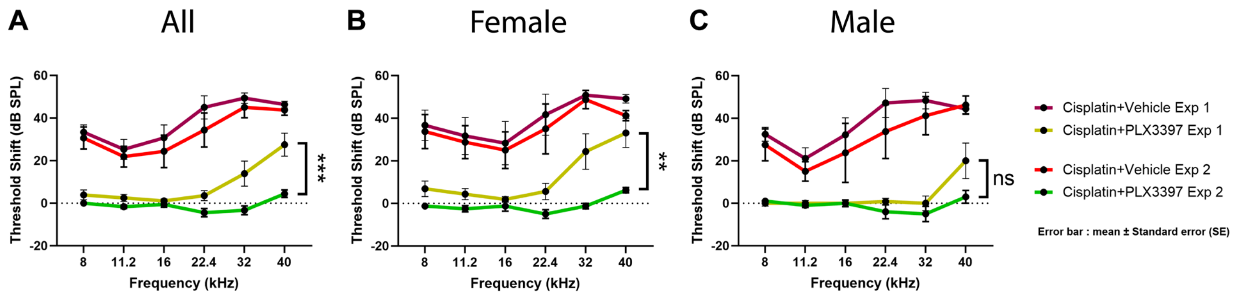
A
All
B
Female
C
Male
Threshold Shift (dB SPL)
Frequency (kHz)
60
40
20
0
-20
8
11.2
16
22.4
32
40
***
**
ns
Cisplatin+Vehicle Exp 1
Cisplatin+PLX3397 Exp 1
Cisplatin+Vehicle Exp 2
Cisplatin+PLX3397 Exp 2
Error bar : mean ± Standard error (SE)

Fig. S12. Sustained macrophage ablation (Experiment 2) provides more robust protection against cisplatin-induced hearing loss compared to macrophage ablation followed by partial repopulation (Experiment 1). Hearing loss was assessed by auditory brainstem responses (ABRs) before (baseline) PLX3397 treatment and after (endpoint) completion of the cisplatin administration protocol. Hearing loss is reported as threshold shifts (the difference between baseline and endpoint ABR thresholds). ABR threshold shifts were assessed and plotted side-by-side for Experiment 1 and Experiment 2, specifically for the two groups, Cisplatin+Vehicle and Cisplatin+PLX3397, across (A) all mice, (B) female mice, and (C) male mice. Statistical analyses were performed using two way-ANOVA with Tukey's multiple comparisons test (main column effect). Experiment 1 (Exp 1): Mean±SEM, n=10-16 mice (4-7F and 6-9M) per experimental group (total 26 mice; 11F and 15M); Experiment 2 (Exp 2): Mean±SEM, n=10-16 mice (4F and 4-5M) per experimental group (total 17 mice; 8F and 9M).

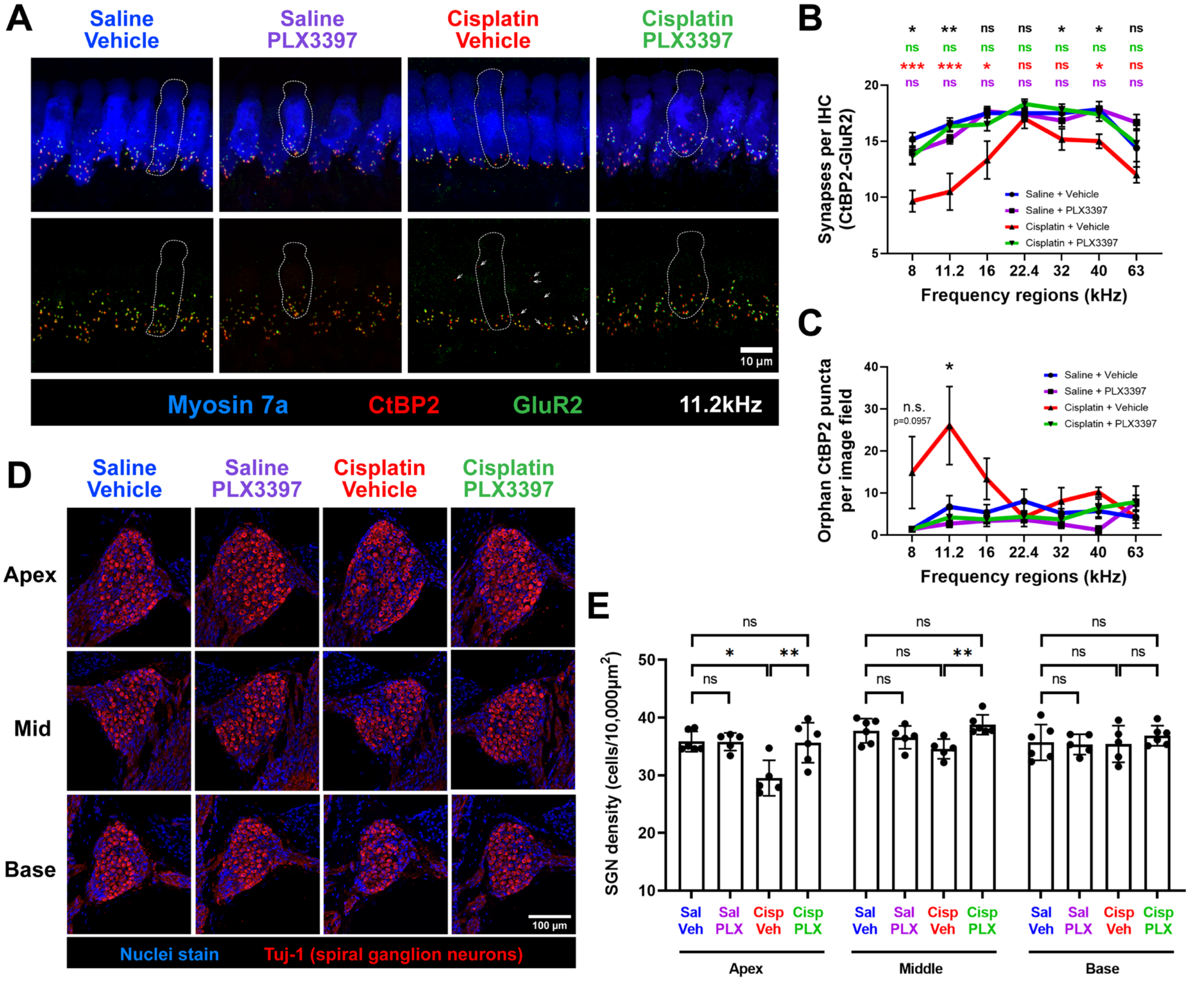
A
Saline Vehicle
Saline PLX3397
Cisplatin Vehicle
Cisplatin PLX3397
10 µm
Myosin 7a
CtBP2
GluR2
11.2kHz
B
Synapses per IHC (CtBP2-GluR2)
Frequency regions (kHz)
8
11.2
16
22.4
32
40
63
Saline + Vehicle
Saline + PLX3397
Cisplatin + Vehicle
Cisplatin + PLX3397
C
Orphan CtBP2 puncta per image field
n.s.
p=0.0957
Frequency regions (kHz)
Saline + Vehicle
Saline + PLX3397
Cisplatin + Vehicle
Cisplatin + PLX3397
D
Saline Vehicle
Saline PLX3397
Cisplatin Vehicle
Cisplatin PLX3397
Apex
Mid
Base
100 µm
Nuclei stain
Tuj-1 (spiral ganglion neurons)
E
SGN density (cells/10,000µm2)
Sal Veh
Sal PLX
Cisp Veh
Cisp PLX
Apex
Middle
Base

Fig. S13. Sustained macrophage ablation protects against cisplatin-induced loss of synapses and SGNs (Experiment 2). (A-C) Cochlear wholemounts were stained for Myosin 7a (blue), CtBP2 (pre-synaptic ribbon), and GluR2 (post-synaptic glutamate receptor). (A) Representative images and quantitative analysis of (B) CtBP2-GluR2 juxtaposed synapses that span 8-63kHz cochlear regions. (A) Orphan CtBP2 pre-synaptic punctae were observed in cisplatin/vehicle-treated mice (white arrows) and (C) quantified. (A) Scale bar, 10 µm. (B-C) Data are shown as Mean±SEM, n=6 cochleae (3F and 3M) per experimental group (total 24 cochleae; 12F and 12M). P values were calculated using one-way ANOVA with Tukey's multiple comparisons test. Statistical comparisons (asterisks or n.s.) are color-coded as outlined in Methods. (D-E) Mid-modiolar cochlear sections were stained for Tuj-1 to visualize and quantify SGNs. Nuclei were stained with Hoechst 33342 (blue). (E) SGNs were quantified and normalized the counts to the areas of Rosenthal's canal to provide SGN density (10,000μm^2) in the apical, mid, and basal regions of the cochlea. (D) Scale bar, 100 µm. (E) Data are shown as Mean±SD, n=5-6 cochleae (2F and 3-4M) per experimental group (total 22 cochleae; 8F and 14M). P values were calculated using one-way ANOVA with Tukey's multiple comparisons test.

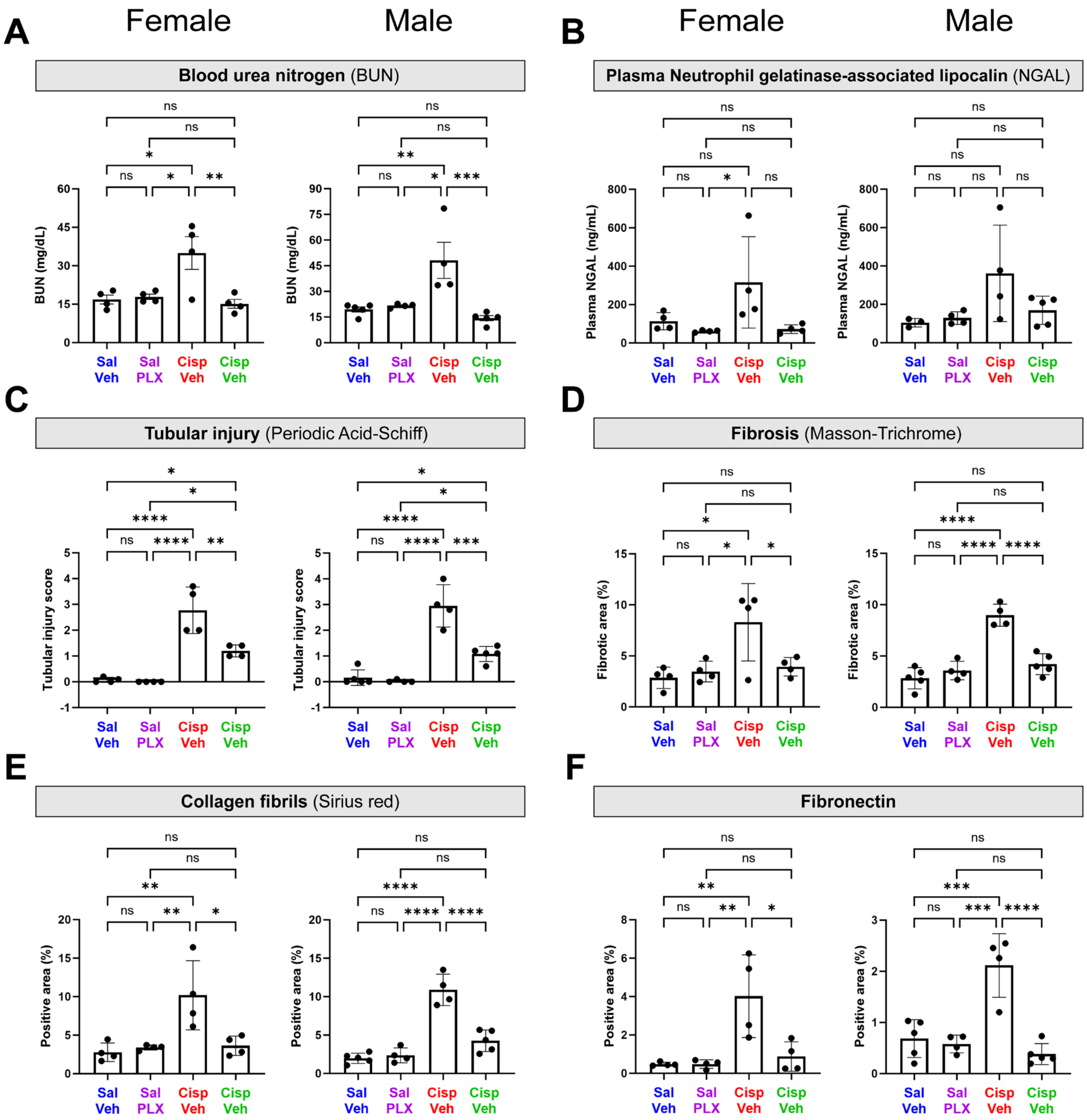
A
Female
Male
Blood urea nitrogen (BUN)
BUN (mg/dL)
B
Female
Male
Plasma Neutrophil gelatinase-associated lipocalin (NGAL)
Plasma NGAL (ng/mL)
C
Tubular injury (Periodic Acid-Schiff)
Tubular injury score
D
Fibrosis (Masson-Trichrome)
Fibrotic area (%)
E
Collagen fibrils (Sirius red)
Positive area (%)
F
Fibronectin
Positive area (%)
Sal Veh
Sal PLX
Cisp Veh
Cisp Veh

Fig. S14. Sustained depletion of macrophages via PLX3397 treatment protects against cisplatin-induced nephrotoxicity in both female and male mice (Experiment 2). (A) Plasma blood urea nitrogen (BUN) levels, (B) neutrophil gelatinase-associated lipocalin (NGAL) levels, (C) tubular injury scores assessed using Periodic Acid-Schiff staining, (D) fibrotic area assessed using Masson-Trichrome staining, (E) cell-associated fibrillar collagen-positive areas assessed by Sirius red staining, and (F) fibronectin-positive areas were evaluated and presented separately for female and male mice. (A-F) Data are expressed as mean±SD, n=8–9 blood and kidney samples (4F and 4-5M) per experimental group (total 34 mice; 16F and 18M). P-values were calculated using one-way ANOVA with Tukey's multiple comparisons test.

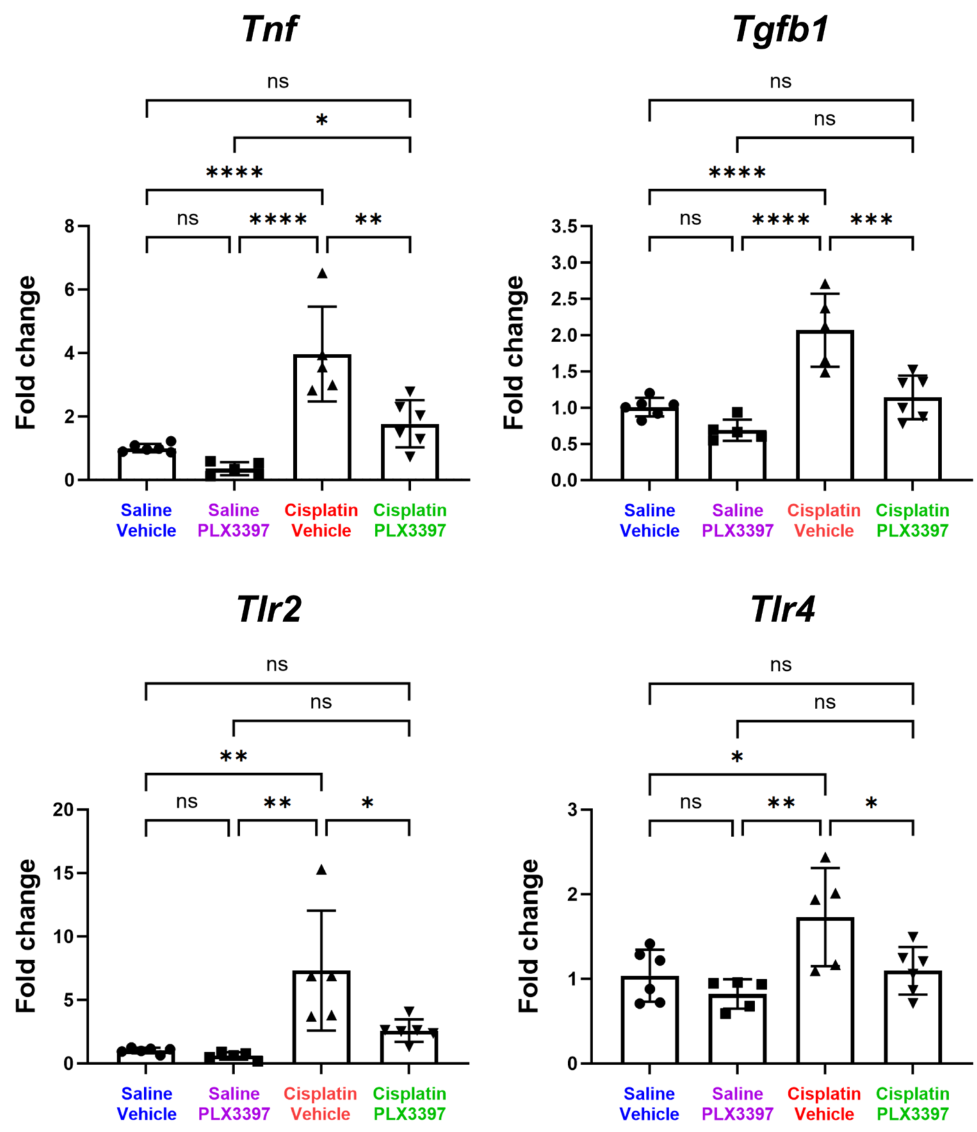
Tnf
ns
*
****
ns
****
**
Fold change
0
2
4
6
8
Saline Vehicle
Saline PLX3397
Cisplatin Vehicle
Cisplatin PLX3397
Tgfb1
ns
ns
****
ns
****
***
Fold change
0.0
0.5
1.0
1.5
2.0
2.5
3.0
3.5
Saline Vehicle
Saline PLX3397
Cisplatin Vehicle
Cisplatin PLX3397
Tlr2
ns
ns
**
ns
**
*
Fold change
0
5
10
15
20
Saline Vehicle
Saline PLX3397
Cisplatin Vehicle
Cisplatin PLX3397
Tlr4
ns
ns
*
ns
**
*
Fold change
0
1
2
3
Saline Vehicle
Saline PLX3397
Cisplatin Vehicle
Cisplatin PLX3397

Fig. S15. Sustained macrophage ablation via PLX3397 treatment prevented cisplatin-induced elevation of cytokines and toll-like receptors (Experiment 2). Total RNA was extracted from the kidney, and the gene expression levels of cytokines (*Tnf* and *Tgfb1*) and toll-like receptors (*Tlr2* and *Tlr4*) were measured using RT-PCR from the four treatment groups: Saline/Vehicle, Saline/PLX3397, Cisplatin/Vehicle, and Cisplatin/PLX3397. The expression levels were normalized to *Gapdh* (internal control) and are expressed relative to Saline/Vehicle-treated group. (A-F) Data are expressed as mean±SD, n=5–6 kidney samples (2-3F and 2-3M) per experimental group (total 22 mice; 11F and 11M). P-values were calculated using one-way ANOVA with Tukey's multiple comparisons test.

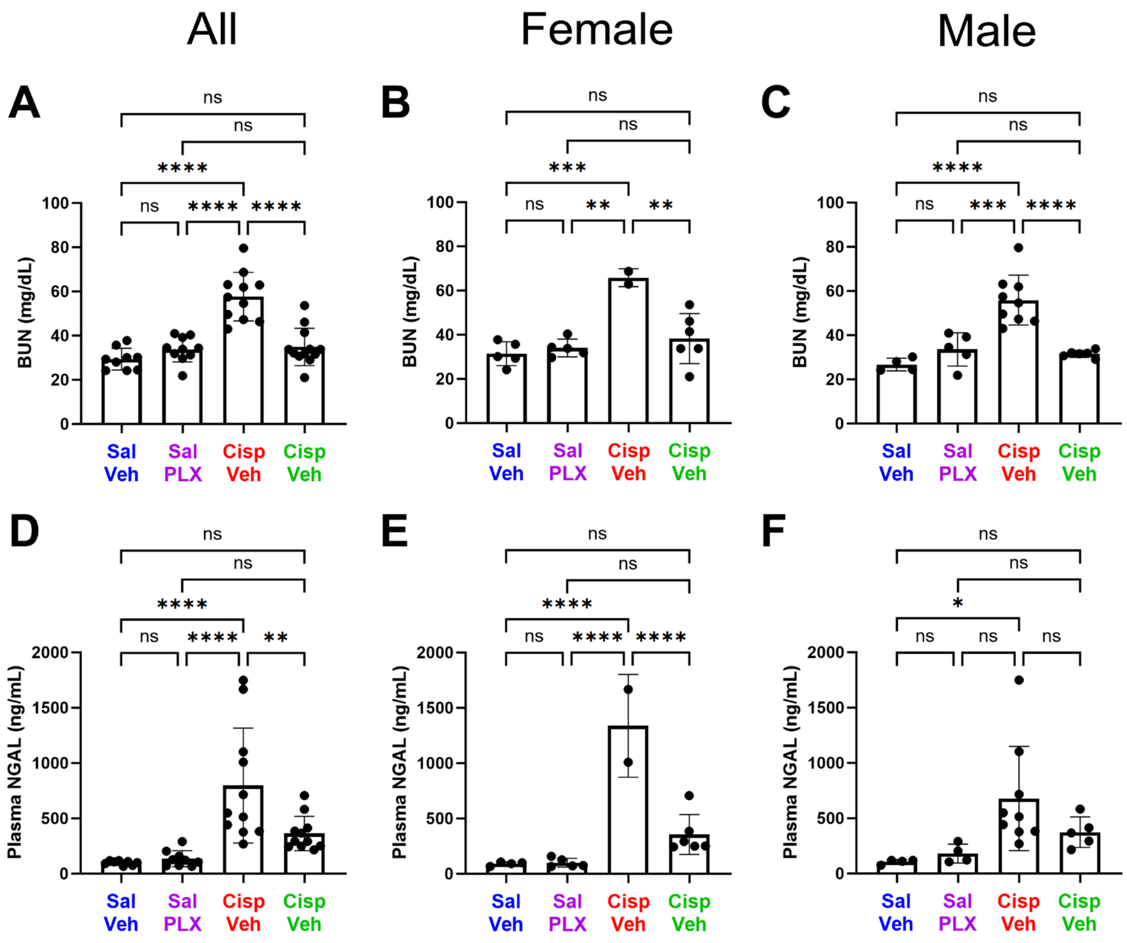
All
Female
Male
A
B
C
D
E
F
BUN (mg/dL)
Plasma NGAL (ng/mL)
ns
****
***
**
*
Sal Veh
Sal PLX
Cisp Veh
Cisp Veh

Fig. S16. Incomplete macrophage ablation via PLX3397 treatment protects against cisplatin-induced nephrotoxicity in both female and male mice (Experiment 1). Blood was collected in heparin-containing syringe from the inferior vena cava and spun down. Plasma was used to measure (A) plasma blood urea nitrogen (BUN) levels and (B) neutrophil gelatinase-associated lipocalin (NGAL) levels to assess kidney injury in (A,D) all, (B,E) female, and (C,F) male mice. (A-F) Data are expressed as mean±SD, n=8–11 blood and kidney samples per experimental group (total 39 mice; 17F and 22M). P-values were calculated using one-way ANOVA with Tukey's multiple comparisons test.

Table S1. Reagents and antibodies used in this study.

Antigen	Clone	Conjugated Fluorophore	Antibody type	Species	Experiment	Company	Catalog #	Accession #
GFP			Purified	Rabbit polyclonal	**IHC** (Kidney)	Abcam	ab290	RRID:AB_303395
GFP			Purified	Chicken IgY	**IF** (Inner ear)	ThermoFisher (Invitrogen)	A10262	RRID:AB_2534023
GFP		AF488	Conjugated	Rabbit polyclonal	**IF** (Inner ear)	ThermoFisher (Invitrogen)	A21311	RRID:AB_221477
Kir4.1 (KCNJ10)			Purified	Rabbit polyclonal	**IF** (Inner ear)	Alamone	APC-035	RRID:AB_2040120
Myosin VIIa			Purified	Rabbit polyclonal	**IF** (Inner ear)	Proteus Bioscience	25-6790	RRID:AB_10015251
Tubulin βIII	Tuj-1		Purified	mouse IgG2a	**IF** (Inner ear)	BioLegend	801213	RRID:AB_2728521
CD31-AF647	MEC13.3	AF647	Conjugated	Rat IgG2a	**IF** (Inner ear)	BioLegend	102515	RRID:AB_2161030
GluR2	6C4		Purified	Mouse IgG2a	**IF** (Inner ear)	EMD millipore	MAB397	RRID:AB_2113875
CtBP2	16/CtBP2		Purified	Mouse IgG1	**IF** (Inner ear)	BD Transduction Laboratories	612044	RRID:AB_399431
Goat anti-rabbit IgG(H+L)		Plus AF405	Secondary Antibody		**IF** (Inner ear)	Invitrogen	A48254	RRID:AB_2890548
Goat anti-chicken IgG(H+L)		AF488	Secondary Antibody		**IF** (Inner ear)	Invitrogen	A11039	RRID:AB_2534096
Goat anti-mouse IgG2a		AF488	Secondary Antibody		**IF** (Inner ear)	Invitrogen	A21131	RRID:AB_141618
Goat anti-mouse IgG1		AF568	Secondary Antibody		**IF** (Inner ear)	Invitrogen	A21124	RRID:AB_141611
Goat anti-rabbit IgG(H+L)		TRITC	Secondary Antibody		**IF** (Inner ear)	SouthernBiotech	4010-03	RRID:AB_2795916
Goat anti-rat IgG(H+L)		AF647	Secondary Antibody		**IF** (Inner ear)	Invitrogen	A21247	RRID:AB_141778
Hoechst 33342			Nuclei Dye		**IF** (Inner ear)	Invitrogen	H3570	
Fixable Viability Dye		eFluor450			**Flow** (spleen)	ThermoFisher (eBioscience)	65-0863-14	
CD45.2	104	BUV395	Conjugated		**Flow** (spleen)	BD Biosciences	564616	RRID:AB_2738867
CD45.2	104	PE-Cy7	Conjugated		**Flow** (spleen)	BioLegend	109829	RRID:AB_1186103
CD11b	M1/70	BV785	Conjugated		**Flow** (spleen)	BioLegend	101243	RRID:AB_2561373
Ly6G	1A8	BUV395	Conjugated		**Flow** (spleen)	BD Biosciences	563978	RRID:AB_2716852
F4/80	BM8	PE	Conjugated		**Flow** (spleen)	BioLegend	123109	RRID:AB_893498
CD3ε	145-2C11	PE	Conjugated		**Flow** (spleen)	BioLegend	100307	RRID:AB_312672
CD19	6D5	PE-Cy7	Conjugated		**Flow** (spleen)	BioLegend	115519	RRID:AB_313654
NK1.1	PK136	APC	Conjugated		**Flow** (spleen)	BioLegend	108709	RRID:AB_313396

IHC = Immunohistochemistry, IF = Immunofluorescence, Flow = Flow cytometry
